# Loss of O-GlcNAc transferase activity impairs the dynamic formation of protective stress granules in ischemic cardiomyocytes

**DOI:** 10.1042/BCJ20250300

**Published:** 2026-09-16

**Authors:** Hannah C. Swimm, Wenxi Zhang, Yashas Mallikarjun, Rohit Satish, Andres J. Medina, Sabrina Lo, Hisham Qureshi, Aidan J. Dunphy, Deepthi Ashok, Agnes Sidor, Olurotimi O. Mesubi, Brian O'Rourke, D. Brian Foster, Natasha E. Zachara, Kyriakos N. Papanicolaou

**Affiliations:** 1 Division of Cardiology, Department of Medicine, Johns Hopkins University School of Medicine, Baltimore, Maryland, U.S.A.; 2 Department of Biophysics and Biophysical Chemistry, Johns Hopkins University School of Medicine, Baltimore, Maryland, U.S.A.; 3 Department of Oncology, Johns Hopkins University School of Medicine, Baltimore, Maryland, U.S.A.

**Keywords:** cardiomyocytes, cytoprotection, G3BP1, Ischemia, O-GlcNAc, Stress Granules

## Abstract

Stress granules are transient biomolecular condensates of proteins and RNA formed during stress. Stress granules support cell survival by sequestering components of the translational machinery, suppressing pro-apoptotic signaling, and conserving cellular resources. O-GlcNAcylation, a dynamic modification of cytoplasmic proteins, is an important regulator of stress responses, including stress-granule assembly. O-GlcNAcylation is protective in acute myocardial stress; however, whether O-GlcNAcylation regulates stress granules to protect cardiomyocytes remains unclear. Here, we investigated whether O-GlcNAcylation regulates stress granule formation in cardiomyocytes exposed to sodium arsenite stress or ischemia/reperfusion (I/R) injury. G3BP1 is an RNA-binding protein that is essential in stress granule formation. Using an EGFP–G3BP1 reporter, we found that OGT, the enzyme responsible for installing O-GlcNAc on target proteins, is necessary for efficient stress-granule formation in cardiomyocytes. Importantly, we identified T268 of G3BP1 as an O-GlcNAcylation site in these cells and demonstrated by mutagenesis its functional importance for stress granule assembly. In primary cardiomyocytes subjected to simulated I/R, stress granules formed transiently during ischemia, and this response was decreased by OGT-knockdown. Similarly, stress granules were transiently detected at the ischemic phase of I/R injury, and their numbers were reduced in OGT-deficient hearts. Furthermore, pretreatment with sodium arsenite before I/R was cardioprotective. Likewise, pretreatment with sodium arsenite protected cells from staurosporine-induced death, and this effect was decreased by OGT knockdown. Together, these findings identify OGT and O-GlcNAcylation as important regulators of stress-granule assembly in cardiomyocytes, define G3BP1 as an O-GlcNAc-modified effector, and demonstrate that stress granules can act as cytoprotective mediators during acute cardiac injury.

## Introduction

O-GlcNAcylation is the reversible addition of O-linked N-acetylglucosamine to serine/threonine residues, serving as a signaling modality that regulates thousands of intracellular proteins [1–3]. O-GlcNAc transferase (OGT) is the enzyme that installs O-GlcNAc on proteins, whereas the O-GlcNAcase (OGA) removes the GlcNAc moiety [4]. O-GlcNAcylation has been shown to coordinate virtually every intracellular process, including transcriptional control [5,6], post-transcriptional RNA splicing [7], ribosome function, translational initiation [8,9], and protein quality control [10–13]. Additionally, O-GlcNAcylation dynamically changes in response to cellular stresses, fine-tuning the aforementioned processes [14–16]. However, the mechanistic details of how O-GlcNAcylation impacts specific cellular stress responses remain incompletely understood.

Cardiac ischemia/reperfusion (I/R) injury stems from the abrupt deprivation and resupply of nutrients and oxygen, resulting in a burst of reactive oxygen species (ROS), ion dysregulation, and mitochondrial Ca^2+^ overload, that strain proteostasis and activate cell-death programs [17–19]. Importantly, short-term elevation of O-GlcNAc is protective in the context of myocardial I/R injury [20–23], buttressed by evidence from knockout mice indicating that OGT protects against heart failure after acute MI [24]. Mechanistically, the protective effect of O-GlcNAcylation in the context of I/R injury has been associated with preservation of mitochondrial membrane potential, improved mitochondrial Ca^2+^ and ROS handling, as well as reduced cytosolic Ca^2+^ release and ER stress [23,25–28]. Nonetheless, the molecular events that impact these pathways and additional cardioprotective events remain ill-defined. During acute stress, non-translating messenger ribonucleoproteins (stalled mRNA–protein assemblies that include ribosomal proteins and translation initiation factors) coalesce into cytoplasmic, membraneless biomolecular condensates called stress granules [29–31]. G3BP1 (Ras-GAP SH3 domain-binding protein 1), a conserved RNA-binding protein, nucleates stress-granule assembly by binding RNA and multimerizing through low-complexity regions [32,33]. Additional RNA-binding proteins, such as TIA1 and Caprin1, act as regulators that tune stress granule assembly and dynamics [34–36]. Early genetic screening showed that OGT was required for efficient stress-granule assembly, and several stress-granule components, including ribosomal proteins and regulatory RNA-binding proteins, undergo O-GlcNAcylation during stress [37,38]. Interestingly, OGT appears to preferentially glycosylate low-complexity, intrinsically disordered regions (IDRs) of proteins [39,40]. These regions mediate weak, multivalent contacts that drive liquid–liquid phase separation, a defining step in stress-granule formation [41,42].

G3BP1 contains an N-terminal domain, termed NTF2L, that is essential in homodimer formation. It includes an acidic domain, a proline-rich (PxxP) segment that serves as an SH3-binding hub, an RNA-recognition motif (RRM) crucial for RNA binding, and an arginine-glycine-rich region (RGG) [43,44]. Within G3BP1, the NTF2L and RRM are predicted to be structured domains, whereas the other regions, including the acidic and PxxP domains, are predicted to have IDRs [45]. During stress, G3BP1 dimers adopt an ‘open’ conformation, and the RRM becomes available for multivalent interactions with RNA molecules to initiate stress granule formation [32,33,46]. In cardiomyocytes, G3BP1 suppresses pre-miR-1 processing and promotes hypertrophic growth [47,48]. Furthermore, G3BP1 has been reported to ameliorate inflammatory and mitochondrial functional readouts in lipopolysaccharide-treated cardiomyocytes [49].

Stress granules support survival during apoptotic signaling and oxidative stress, although persistent or abnormal assemblies can be deleterious, particularly in neurodegenerative settings [50–52]. Nevertheless, in acute cerebral events, stress granules form transiently during ischemia and are associated with neuroprotection [53,54]. In cardiac cells, stress granules are reported to form transiently in response to acute pacing and have been associated with mitigation of Ca^2+^ overload, oxidative stress, and doxorubicin toxicity [55,56]. Nevertheless, key signaling modalities and mechanisms regulating stress granule formation in cardiac myocytes remain unclear. Here, we hypothesized that O-GlcNAcylation governs stress-granule assembly in cardiomyocytes. We demonstrate that O-GlcNAcylation regulates assembly and identify G3BP1 as a key O-GlcNAc-sensitive effector. Employing cell and mouse models of acute cardiac injury, we show that stress granules form during ischemia and resolve with reperfusion. Finally, we demonstrate that preemptive formation of stress granules confers cytoprotection against cell-death stimulation.

## Results

### Pharmacologic inhibition or genetic knockdown of OGT reduces SG formation

O-GlcNAcylation has been implicated in regulating cellular stress responses, yet its role in modulating stress granule (SG) formation in cardiac myocytes remains unclear. First, we confirmed that O-GlcNAcylation is enriched in cardiomyocyte SGs. To that end, we treated primary neonatal rat ventricular myocytes (NRVMs) with the SG inducer sodium arsenite and performed immunofluorescence staining using the mouse monoclonal anti-O-GlcNAc antibody RL2 together with various SG markers (G3BP1, eIF4G1, PABPC1, and TIA1). Indeed, O-GlcNAc showed strong immunoreactivity in subcellular granule formations that were positive for the chosen SG markers (Supplementary Figure S1). The specificity of RL2 immunoreactivity was further validated by preincubating the antibody with excess free GlcNAc, which markedly reduced O-GlcNAc staining (Supplementary Figure S2A,B). Moreover, immunostaining with an independent anti-O-GlcNAc antibody (Rabbit multimAb) revealed a similar pattern of O-GlcNAc accumulation in SA-induced G3BP1-positive puncta (Supplementary Figure S2C).

**Figure 1:**
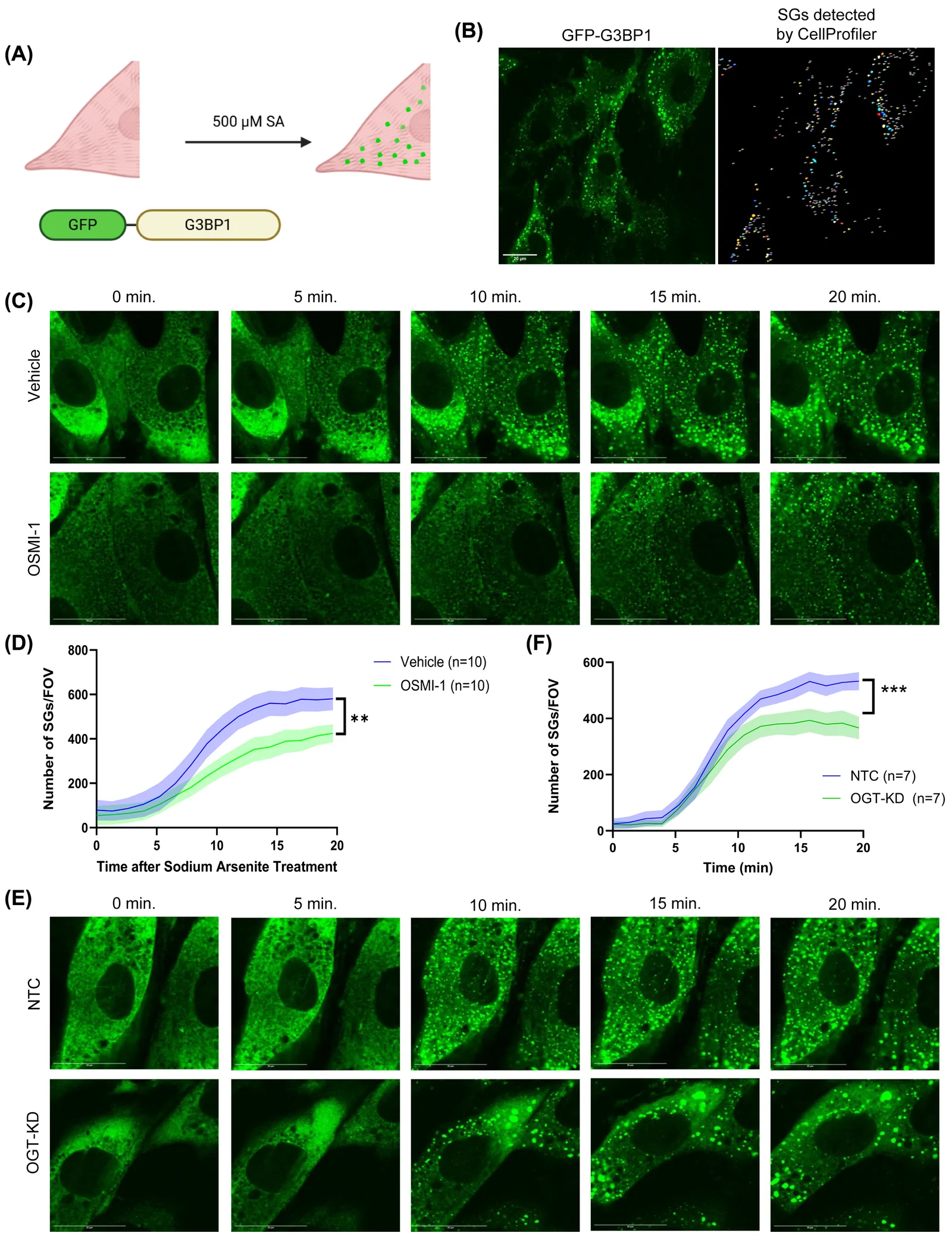
Inhibition and knockdown of OGT reduce SG formation (**A**) Schematic showing formation of GFP-G3BP1-positive granules in H9c2 cells treated with sodium arsenite (SA). (**B**) Representative of images of GFP-G3BP1 signal in H9c2 cells 20 minutes following treatment with 500 μM sodium arsenite (left). Images were acquired using an FV3000 Olympus confocal microscope using the 100× objective. Scale bars: 20 μm. Regions of interest (ROIs) identified as SGs by CellProfiler using Robust Thresholding (right). (**C**) Representative images of GFP-G3BP1 signal in vehicle-treated and 25 μM OSMI-1-treated H9c2 cells in minutes following treatment with 500 μM sodium arsenite. Scale bars: 20 μm. (**D**) Number of stress granules/field of view (SGs/FOV) identified in images of vehicle-treated H9c2 cells compared with 25 μM OSMI-1-treated H9c2 cells in regions of comparable confluence (*n* = 10 replicates per group). Statistical comparisons were performed using two-way repeated measures ANOVA (** *P* = 0.0014). (**E**) Representative images of GFP-G3BP1 signal in NTC and OGT knockdown H9c2 cells in minutes following treatment with 500 μM sodium arsenite. Scale bars: 20 μm. (**F**) Number of SGs/FOV identified in images of NTC H9c2 cells (blue) compared with OGT-KD H9c2 cells (green) in regions of comparable confluence (*n* = 7 replicates per group). Statistical comparisons were performed using two-way repeated measures ANOVA (*** *P* = 0.0002).

Next, we sought to determine the impact of O-GlcNAcylation on the kinetics of SG formation. To address this question, we assessed the rates of SG formation in H9c2 cardiomyocytes. Cells were transduced to express EGFP–G3BP1, enabling real-time visualization and quantification of SG assembly (Figure 1A). After adenoviral transduction (MOI = 50, 24 h), cells were treated with vehicle (DMSO) or the OGT inhibitor OSMI-1 (25 μM, 6 h), which effectively reduces O-GlcNAcylation levels (Supplementary Figure S3A–C). The cells were then challenged with sodium arsenite (SA, 500 μM) to induce SG formation (Figure 1A). SG formation was monitored over a 20-min period with a confocal microscope (Olympus FV3000RS, enabled with z-drift compensation system, ∼1 image/min). The number of SGs per image was quantified by the CellProfiler program using the Robust Thresholding feature (Figure 1B). Using this approach, we found that the sigmoidal curve of SG formation was downwardly shifted in cells treated with OSMI-1, concomitant with significantly fewer SGs compared with vehicle-treated controls (Figure 1C,D, *P* < 0.01). To corroborate these findings using an independent genetic approach, we targeted OGT via siRNA-mediated knockdown (20 nM, 48 h). We confirmed reduced OGT protein abundance and global O-GlcNAc levels by the siRNA used to generate the OGT-KD condition (siOGT #1), as well as with two additional independent OGT-targeting siRNAs (Supplementary Figure S4). Control (NTC) and OGT-KD cells were then analyzed using the same real-time SG formation assay described above. Consistent with the results obtained using OGT inhibition (OSMI-1), OGT knockdown reduced SG formation (Figure 1E,F, *P* < 0.05).

**Figure 2:**
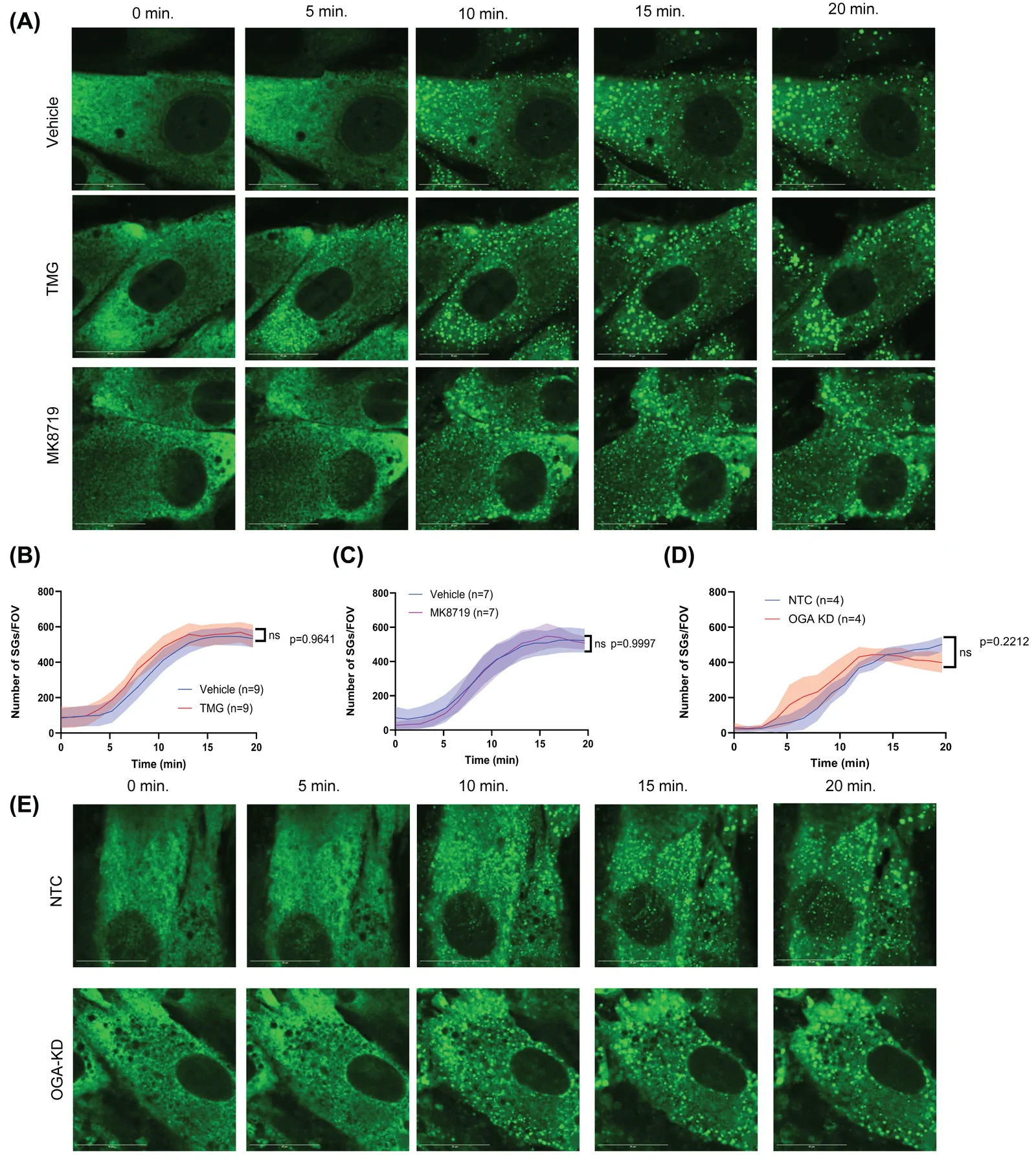
Inhibition and knockdown of OGA does not impact SG formation (**A**) Representative images of GFP-G3BP1 signal in vehicle-treated, TMG-treated (25 μM), and MK-8719-treated (25 μM) H9c2 cells in minutes following treatment with 500 μM sodium arsenite. Scale bars: 20 μm. (**B**) Number of SGs/FOV identified in images of vehicle-treated H9c2 cells compared with 25 μM TMG-treated H9c2 cells in regions of comparable confluence (*n* = 9 replicates per group). Statistical comparisons were performed using two-way repeated measures ANOVA (*P* = 0.9641). (**C**) Number of SGs/FOV identified in images of vehicle-treated H9c2 cells compared with 25 μM MK-8719-treated H9c2 cells in regions of comparable confluence (*n* = 7 replicates per group). Statistical comparisons were performed using two-way repeated measures ANOVA (*P* = 0.9997). (**D**) Number of SGs/FOV identified in images of NTC H9c2 cells compared with OGA-KD H9c2 cells in regions of comparable confluence (*n* = 4 replicates per group). Statistical comparisons were performed using two-way repeated measures ANOVA (*P* = 0.2075). (**E**) Representative images of EGFP-G3BP1 signal in NTC (top) and OGA-knockdown (bottom) H9c2 cells in minutes following treatment with 500 μM sodium arsenite. Scale bars: 20 μm.

Next, we examined whether inhibiting OGA, the enzyme that catalyzes the removal of O-GlcNAc, impacts SG formation. To that end, we treated cells with vehicle or the OGA inhibitor thiamet-G (TMG, 25 μM, 6 h), which strongly elevated O-GlcNAc levels while modestly affecting OGT and OGA protein abundance (Supplementary Figure S5A–C). Unlike the studies with OGT inhibition, we found that SG formation was not significantly changed by OGA inhibition (Figure 2A,B). Similarly, use of an alternative OGA inhibitor, MK8719 (25 μM, 6 h, Figure 2A,C; Supplementary Figure S5D–F), or siRNA-mediated knockdown of OGA (20 nM, 48 h; Supplementary Figure S5G–I) had no impact on the formation of SGs (Figure 2D,E).

### Differential recruitment of OGT and OGA enzymes on stress granules upon sodium arsenite treatment

**Figure 3:**
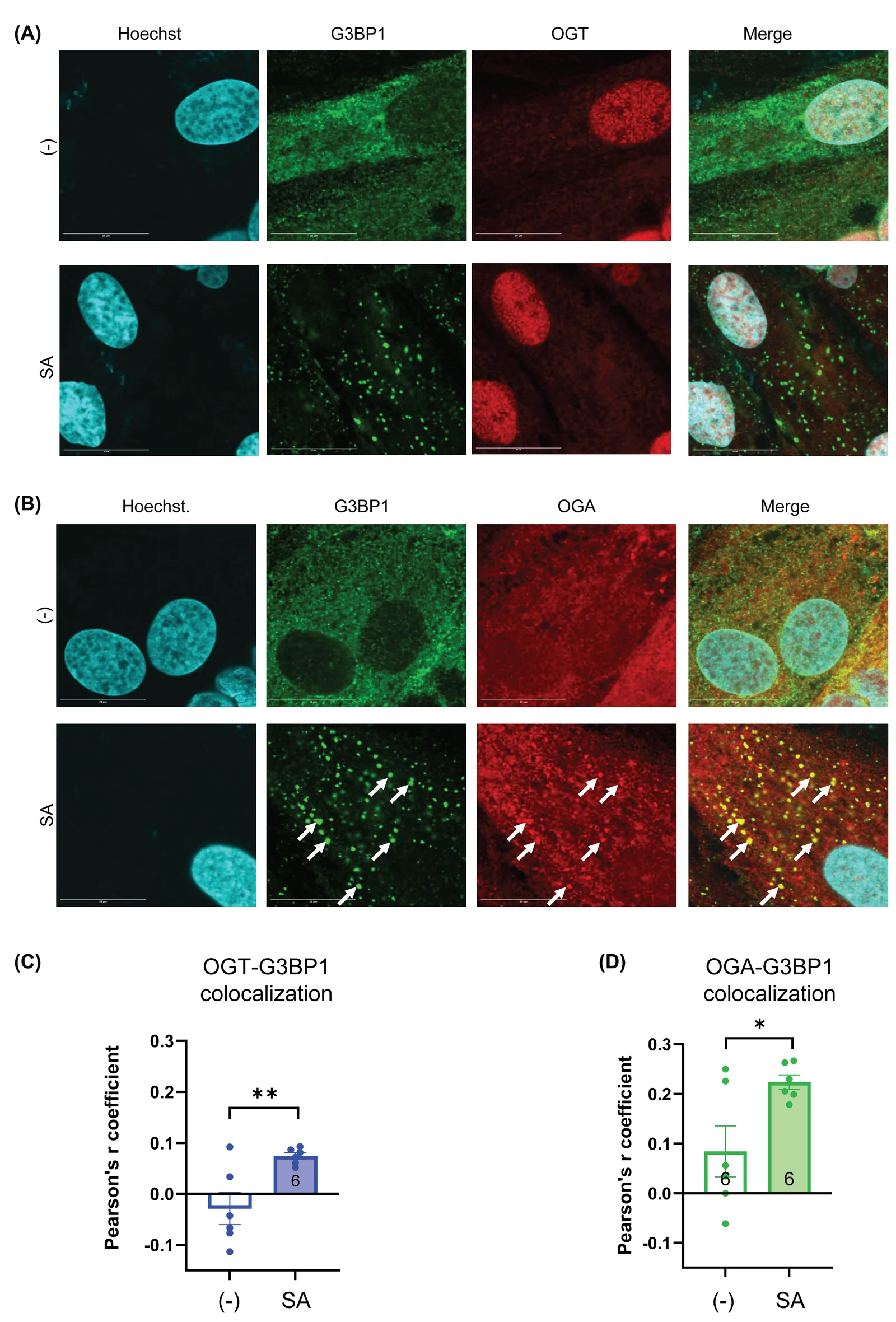
OGA colocalizes with G3BP1 in SGs, but OGT does not (**A**) Fluorescent immunostaining for OGT and G3BP1 with nuclear Hoechst staining of H9c2 in the presence and absence of 500 μM sodium arsenite. Scale bars: 20 μm. (**B**) Fluorescent immunostaining for OGA and G3BP1 with nuclear Hoechst staining of H9c2 in the presence and absence of 500 μM sodium arsenite. Scale bars: 20 μm. Arrows indicate colocalization of OGA and G3BP1 in SGs. (**C**) Quantification of colocalization between G3BP1 and OGT in H9c2 cells in the absence (*n* = 6) or presence (*n* = 6) of 500 μM sodium arsenite reported as Pearson’s correlation coefficient in cytoplasmic ROIs identified by Cell Profiler. Statistical comparisons were performed using a two-tailed t-test (** *P* = 0.0091). (**D**) Quantification of colocalization between G3BP1 and OGA in H9c2 cells in the absence (*n* = 6) or presence (*n* = 6) of 500 μM sodium arsenite reported as Pearson’s correlation coefficient in cytoplasmic ROIs identified by CellProfiler. Statistical comparisons were performed using a two-tailed t-test (* *P* = 0.0259).

Building on the functional data above, we next examined the spatial distribution of enzymes OGT and OGA relative to SGs using immunohistochemistry. To that end, H9c2 cells treated with sodium arsenite (500 μM, 30 min) were subsequently fixed and stained for the SG marker G3BP1 with either OGT or OGA. As shown in Figure 3A, the distribution of OGT was spatially distinct from that of G3BP1, with most of the OGT signal detected in the nucleus. Moreover, OGT and G3BP1 signals showed little spatial overlap either at baseline or after sodium arsenite treatment. By contrast, OGA showed a weak colocalization with G3BP1 at baseline and, upon sodium arsenite treatment, a clear colocalization with G3BP1 within SGs (Figure 3B). Pearson’s correlation analysis within cytoplasmic ROIs yielded values near zero for OGT–G3BP1 under both basal (–0.029) and sodium arsenite-treated (+0.074) conditions, consistent with the absence of visual co-localization (Figure 3C). In contrast, OGA–G3BP1 correlations increased from 0.084 at baseline to 0.224 after sodium arsenite treatment (*P* < 0.05), reflecting a shift toward positive correlation. It is important to note that, while a strong colocalization is visible between OGA and G3BP1 within SGs, the overall r values remained in the weak range (<0.3) due to the substantial diffuse cytoplasmic OGA signal outside SGs, as it can be assessed visually (Figure 3B). Taken together, these results show that the spatial distribution of the two O-GlcNAc cycling enzymes changes upon SG formation and OGA can be acutely recruited to SGs. However, given the earlier findings that OGA is dispensable in our assay of SG formation, these data suggest that enzyme localization alone does not predict their respective contribution to SG dynamics.

### Protein enrichment and mass spectrometry identify site of O-GlcNAcylation on G3BP1

**Figure 4:**
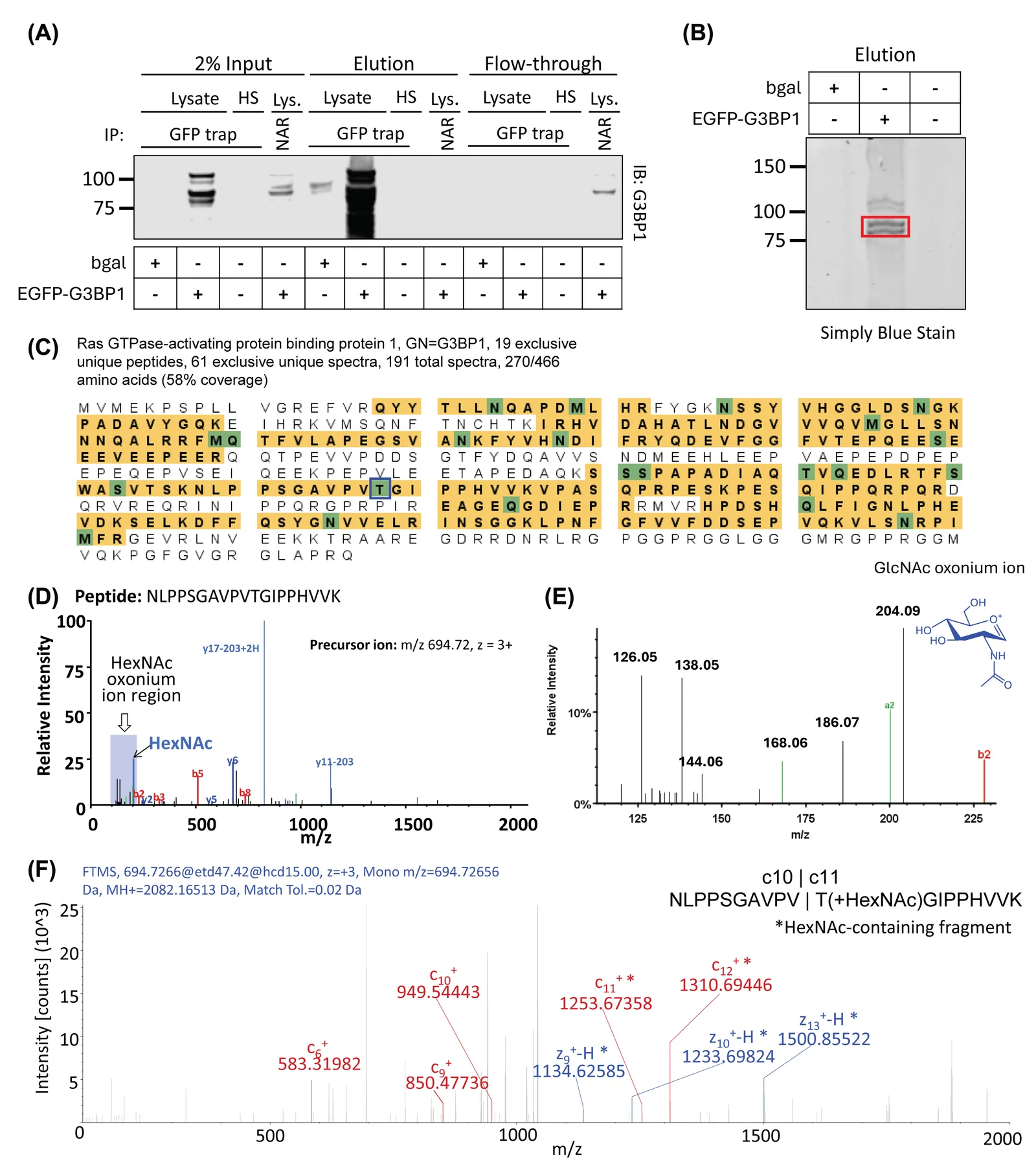
Immunoprecipitation and mass spectrometric analysis of O-GlcNAcylation on G3BP1 (**A**) Representative immunoprecipitation (IP) of EGFP–G3BP1 from H9c2 cell lysates using GFP-Trap resin. HS = harvesting solution (serves as negative control to lysates); NAR = neutral agarose resin control (unconjugated agarose beads used for pre-clearing and as IP negative control). (**B**) Coomassie Brilliant Blue–stained Bis–Tris gradient gel of the GFP-Trap eluates from the three experimental groups (β-gal, EGFP–G3BP1, HS). The EGFP–G3BP1 signal appeared as a distinct doublet at ∼79 kDa, which was excised for LC–MS/MS analysis (red box). (**C**) Amino acid sequence of human G3BP1 showing tryptic peptides unequivocally identified by LC–MS/MS (yellow). Amino acids found to have fixed or variable modifications are shown in green. The identified O-GlcNAcylation site is boxed in blue. Sequence coverage was calculated relative to the UniProt reference sequence Q13283 (RefSeq: NM_005754.3). (**D**) Representative HCD MS/MS spectrum of a precursor ion corresponding to the O-GlcNAc–modified G3BP1 peptide NLPPSGAVPVTGIPPHVVK. The precursor ion was assigned as m/z 694.72 with charge state 3+ and mass error −0.61 ppm. Prominent b- and y-fragment ions are annotated. The shaded low-m/z region highlights diagnostic GlcNAc oxonium ions and is shown enlarged in (E). Fragment ions annotated with −203 indicate neutral loss of GlcNAc. (**E**) Expanded view of the low-m/z region from (D) showing O-GlcNAc diagnostic fragment ions, including m/z 126.055, 138.055, 144.065, 168.066, 186.076, and 204.087. (**F**) Representative EThcD MS/MS spectrum of the G3BP1 peptide NLPPSGAVPVTGIPPHVVK supporting localization of the O-GlcNAc modification to T268. For clarity, selected site-informative c- and z-type fragment ions are annotated. The observed unmodified c6/c9/c10 ions contain the upstream S262 but exclude T268, whereas the observed c11/c12 ions include T268 and correspond to HexNAc-containing fragments. Complementary HexNAc-containing z-type ions further support assignment of the modification to T268. Asterisks indicate HexNAc-containing fragments, and the vertical divider in the peptide sequence indicates the c10/c11 boundary.

To gain a further mechanistic understanding of the impact of O-GlcNAcylation on SG formation, we hypothesized that G3BP1, the central mediator of SG formation, is O-GlcNAcylated. To that end, cells were transduced with β-gal– or EGFP–G3BP1–expressing adenoviruses, harvested 24 h later, and subjected to immunoprecipitation to enrich for EGFP–G3BP1. Fractions corresponding to input, eluate, and flow-through were collected for the following IP groups: β-gal, EGFP–G3BP1, harvesting solution (HS), and lysate + neutral agarose resin (NAR) (Figure 4A). Immunoblotting for G3BP1 revealed strong enrichment of EGFP–G3BP1 in the eluate from GFP-Trap IP, with the signal markedly higher than that in the 2% input and undetectable in the flow-through, indicating near-complete capture. In contrast, incubation of EGFP–G3BP1 lysate with neutral agarose resin (lysate + NAR) yielded little to no signal in the eluate and detectable protein in the flow-through. Together, these results confirm the high efficiency of the pulldown that enriches EGFP–G3BP1 (Figure 4A). The enriched material (∼90%) from the three groups (β-gal, EGFP–G3BP1, HS) was resolved by SDS–PAGE and stained with Coomassie Brilliant Blue, revealing a prominent band at the expected molecular weight of EGFP–G3BP1 (∼79 kDa) that appeared as a doublet (Figure 4B). A second, higher-molecular-weight doublet (>100 kDa) was also observed. The origin of these doublets is unclear but may reflect post-translationally modified or differently processed forms of the protein. Mass spectrometry analysis was performed on the ∼79 kDa doublet, which corresponds to the predicted size of EGFP–G3BP1 (Figure 4B).

The 79 kDa band was excised, subjected to in-gel tryptic digestion, and peptides were analyzed by LC–MS/MS using EThcD strategy. In this approach, HCD fragmentation that results in oxonium ions diagnostic of O-GlcNAc triggers additional ETD fragmentation of the same precursor ion. This workflow provided 58% sequence coverage of G3BP1 (Figure 4C). Upon examination of the MS/MS spectra, we identified evidence for O-GlcNAcylation on G3BP1. Figure 4D shows a representative HCD spectrum of peptide (NLPPSGAVPVTGIPPHVVK) with b/y fragment ion series, together with a signal at m/z 204.087, corresponding to the GlcNAc oxonium ion. Closer inspection of the lower m/z range (Figure 4D, shaded region), shown in greater detail in Figure 4E, revealed a cluster of O-GlcNAc diagnostic fragment ions (m/z 126.055, 138.055, 144.065, 168.066, 186.076), supporting the presence of O-GlcNAc on the identified peptide. To identify the precise residue modified by O-GlcNAc, we examined a collection of ETD spectra for that peptide (Supplementary Table S1). This analysis revealed the presence of high confidence peptide-spectral matches (PSMs) for peptide NLPPSGAVPVTGIPPHVVK (Supplementary Table S2). The spectra contained multiply charged precursor ions with neutral loss of GlcNAc, as well as site-informative c- and z-type fragment ions. Unmodified c9/c10 ions containing the upstream S but excluding T268, together with HexNAc-containing c11/c12 ions that include T268 and complementary HexNAc-containing z-type ions, supported localization of the O-GlcNAc modification to T268 (Figure 4F).

To complement the mass spectrometry analysis of GFP-tagged G3BP1, we next asked whether endogenous G3BP1 could be detected among metabolically labeled O-GlcNAc-modified proteins in a human cardiomyocyte model. To this end, we used a bioorthogonal metabolic-labeling strategy in hiPSC-derived cardiomyocytes expressing UAP1-F383G, an engineered enzyme that facilitates processing of click-compatible GlcNAc analogs (Supplementary Figure S6A). UAP1-F383G-expressing or control cells were incubated with the alkyne-containing GlcNAc analog 1HexGlcNAlk, after which labeled proteins were conjugated to biotin by click chemistry and enriched by streptavidin pulldown (Supplementary Figure S6B). Expression of UAP1-F383G in transduced cells was confirmed by blotting for the myc tag (Supplementary Figure S6C), while global streptavidin blotting confirmed increased click-biotinylated protein signal in UAP1-F383G-expressing cells (Supplementary Figure S6D). Immunoblotting for G3BP1 in streptavidin pull-down samples indicated that a detectable subset of endogenous G3BP1 was enriched from UAP1-F383G-expressing cells (Supplementary Figure S6E). Taken together, these findings provide orthogonal support for G3BP1 as an O-GlcNAc-modified protein in cardiomyocytes.

### G3BP1 T268 supports efficient stress granule formation

**Figure 5:**
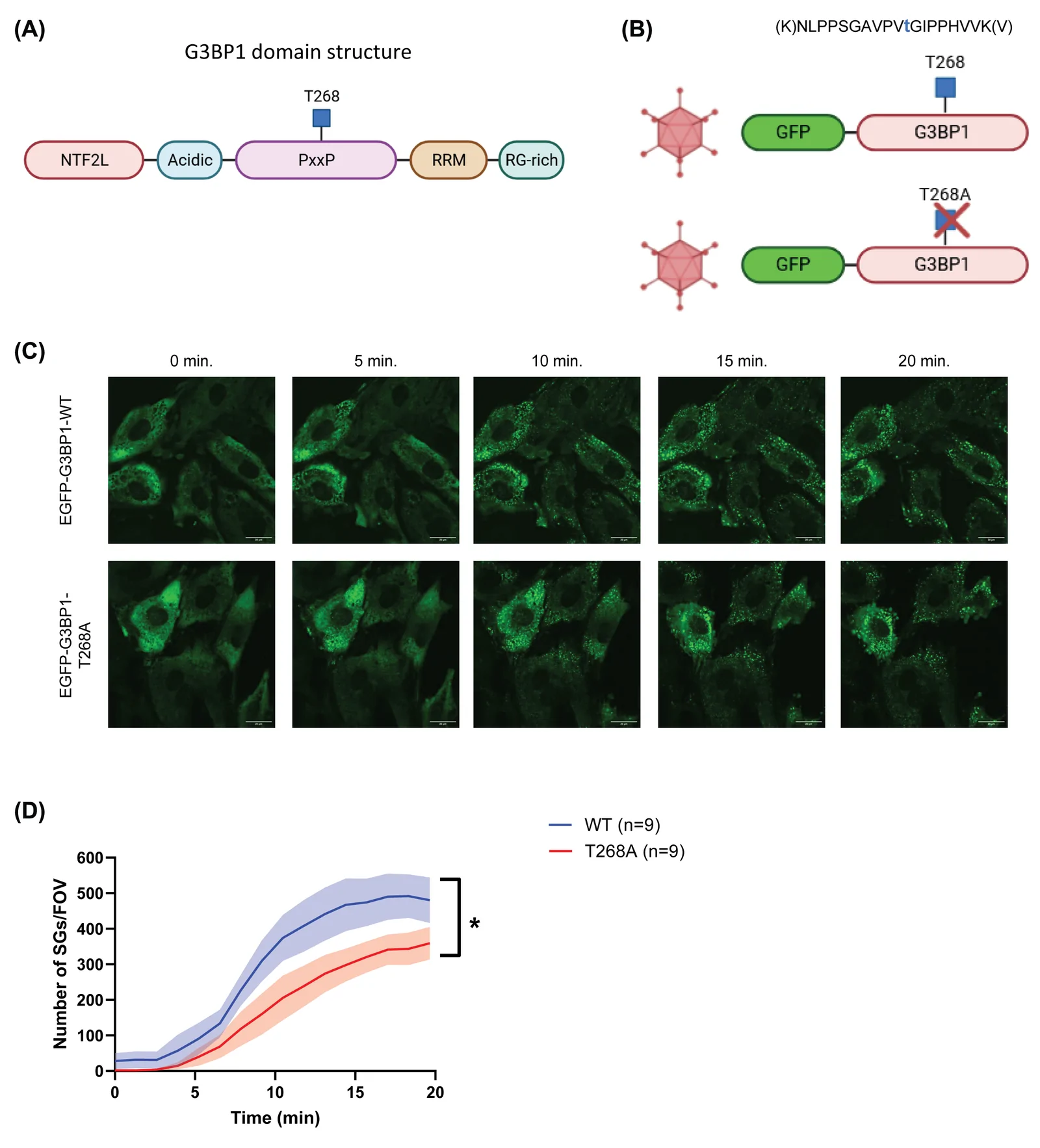
Design of T268A G3BP1 mutant and assessment of its impact on SG formation (**A**) Schematic representation of G3BP1 domain organization, showing the NTF2L domain, acidic domain, PxxP motif–rich region, RRM, and RG-rich domain. The O-GlcNAc–modified threonine residue identified by mass spectrometry (T268) is indicated as the blue square within the PxxP domain. (**B**) Diagrams depicting the EGFP-tagged WT and T268A mutant G3BP1 constructs used in these experiments. Proteins were expressed in cells via adenoviral transduction for functional characterization. (**C**) Representative images of GFP signal in H9c2 cells transduced with viruses expressing WT or T268A mutant G3BP1. Time after addition of 500 μM is indicated as 0, 5, 10, 15, and 20 min. Scale bars: 20 μm. (**D**) Number of SGs/FOV identified in images of cells expressing WT or T268A mutant EGFP-G3BP1 (*n* = 9 replicates per group). The solid lines represent the mean SG number in the WT or T268A groups, and the shaded areas represent ± standard deviation. Statistical comparisons were performed using two-way repeated measures ANOVA (* *P* = 0.0429).

G3BP1 is a modular protein containing five well-characterized domains: an NTF2-like (NTF2L) domain, a central acidic domain, a PxxP motif–rich region, an RNA recognition motif (RRM), and a C-terminal RG-rich domain (Figure 5A). To examine whether O-GlcNAcylation at T268 influences G3BP1 function, we generated a T268A point mutant, replacing the hydroxyl-containing threonine with alanine to prevent O-GlcNAc attachment (Figure 5B). Next, wild-type (WT) and T268A EGFP-G3BP1 versions were delivered to cells via adenoviral transduction, enabling side-by-side assessment of SG assembly in response to sodium arsenite treatment. Real-time confocal live-cell imaging revealed that T268A-expressing cells had significantly lower numbers of SGs compared with WT-expressing cells (Figure 5C). This was also evident from the aggregate data showing the significant rightward and downward shift of the T268A curve (Figure 5D, *P* < 0.05). Taken together, these results suggest that O-GlcNAcylation at T268 plays an important role in SG assembly, consistent with the slower kinetics observed upon OGT targeting (Figure 1).

To further assess G3BP1 O-GlcNAcylation, we performed pulldown experiments from cells expressing WT or T268A G3BP1. WT and T268A G3BP1 were efficiently recovered in the eluate fractions, with minimal signal remaining in the flow-through and little corresponding signal in the β-gal control sample (Supplementary Figure S7A). The same samples were sequentially blotted on the same membrane for O-GlcNAc and G3BP1 using two reciprocal antibody combinations: RL2, a mouse anti-O-GlcNAc antibody, together with rabbit anti-G3BP1; and a rabbit anti-O-GlcNAc multiMAb together with mouse anti-G3BP1 (Supplementary Figure S7B,C). In both approaches, the O-GlcNAc signal overlapped with the G3BP1 band (arrows, Supplementary Figure S7B,C), supporting G3BP1 O-GlcNAcylation and being consistent with the MS/MS and bioorthogonal metabolic-labeling data. O-GlcNAc signal was also detected at the T268A G3BP1 band, indicating that mutation of the functionally important T268 site did not eliminate the total O-GlcNAc signal detected by immunoblot.

Using the same pulldown approach, we next examined whether acute sodium arsenite stress altered the total O-GlcNAc signal detected on G3BP1. WT G3BP1 was recovered from untreated cells and from cells treated with sodium arsenite for 30 or 60 min (Supplementary Figure S7D). Sequential immunoblotting of the corresponding eluates for O-GlcNAc and G3BP1 did not reveal an apparent increase in the O-GlcNAc signal overlapping with the G3BP1 band upon sodium arsenite treatment at either time point (arrow, Supplementary Figure S7E).

### Stress granule formation in ischemic NRVMs and its modulation by OGT depletion

**Figure 6:**
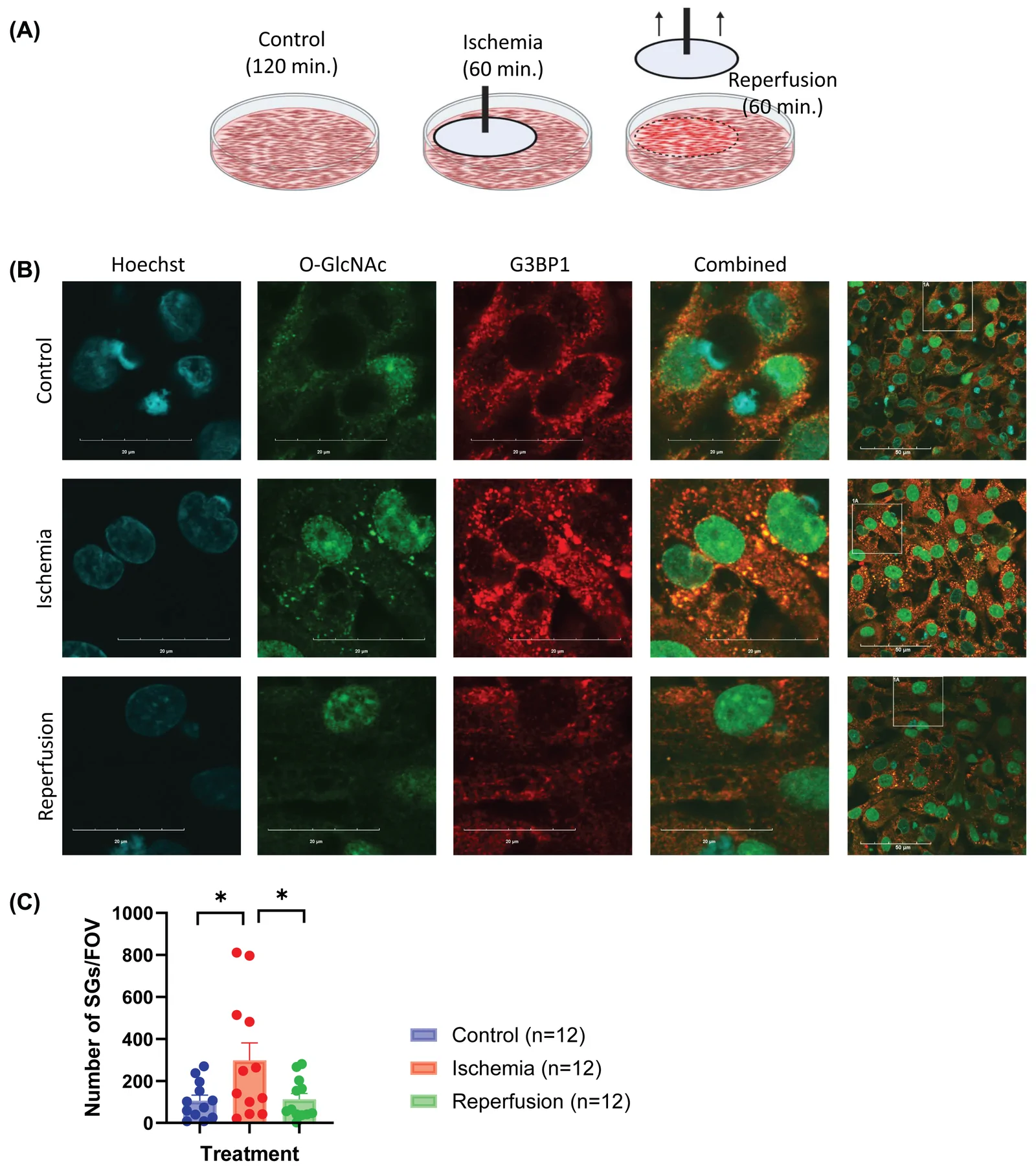
Ischemia induces O-GlcNAc–containing SGs in NRVMs, which dissipate during reperfusion (**A**) Schematic illustration of the coverslip-based procedure used to simulate ischemia and reperfusion injury in NRVMs. Each protocol lasted 120 min and included three experimental groups: *Control*, cells maintained in Tyrode’s solution for 120 min; *Ischemia*, cells subjected to 60 min of glass coverslip application to restrict oxygen and nutrient exchange; and *Reperfusion*, cells subjected to 60 min of ischemia followed by 60 min of recovery after coverslip removal. At the conclusion of each condition, cells were fixed and processed for immunohistochemistry. (**B**) Representative immunofluorescence images showing the three experimental conditions (Control, Ischemia, and Reperfusion). Hoechst staining (blue) marks nuclei, anti-O-GlcNAc staining (green) highlights O-GlcNAcylated proteins, and anti-G3BP1 punctate staining (red) visualizes stress granules. The merged panels demonstrate punctate G3BP1-positive granules, with O-GlcNAc signal frequently overlapping these foci during ischemia. A lower-magnification FOV is included to illustrate the overall monolayer morphology. Images were acquired using an Olympus FV3000RS confocal microscope with a 100× oil-immersion objective. Scale bars: 20 μm (zoomed-in panels); 50 μm (overview images). (**C**) SGs/FOV were quantified across three experimental groups (Control, Ischemia, and Reperfusion) using CellProfiler with robust thresholding. For each condition, 12 independent image fields were analyzed. Statistical comparisons were performed using one-way ANOVA followed by Tukey’s post-hoc test (*P < 0.05; ns, not significant).

To address the broader question of whether cardiomyocyte pathophysiologic injury, such as I/R, is associated with SG formation and how the O-GlcNAcylation system contributes in this context, we employed an established *in vitro* model of cardiac I/R injury [57]. In this approach, a sterile glass coverslip is gently placed over a monolayer of NRVMs, creating a transient barrier that limits oxygen and nutrient exchange with the culture medium. Removal of the coverslip then restores access, mimicking reperfusion injury. (Figure 6A). This model produces three experimental groups (Control, Ischemia, and Reperfusion). To investigate SG dynamics across these stages, we performed immunostaining for G3BP1. In parallel, we also stained for O-GlcNAc to monitor how O-GlcNAcylation is spatially redistributed relative to SGs in the context of cellular I/R (Figure 6B). Immunofluorescence analysis revealed a statistically significant increase in number of SGs in NRVMs that underwent ischemic injury compared with control-treated NRVMs incubated in Tyrode’s buffer for 120 min (Figure 6C, *P* < 0.05). Interestingly, NRVMs that underwent reperfusion had significantly lower number of SGs than ischemic NRVMs (Figure 6C, *P* < 0.05). Taken together, these data suggest that SGs form in NRVMs during ischemia and dissipate during reperfusion. We also observed the overlap between O-GlcNAc staining and punctate G3BP1 signal in ischemic NRVMs, suggesting that O-GlcNAcylated proteins associate with SGs during ischemic stress. This pattern was further confirmed using a second anti-O-GlcNAc antibody, which revealed similar O-GlcNAc immunoreactivity in G3BP1-positive SGs following coverslip-induced ischemic stress (Supplementary Figure S8).

**Figure 7:**
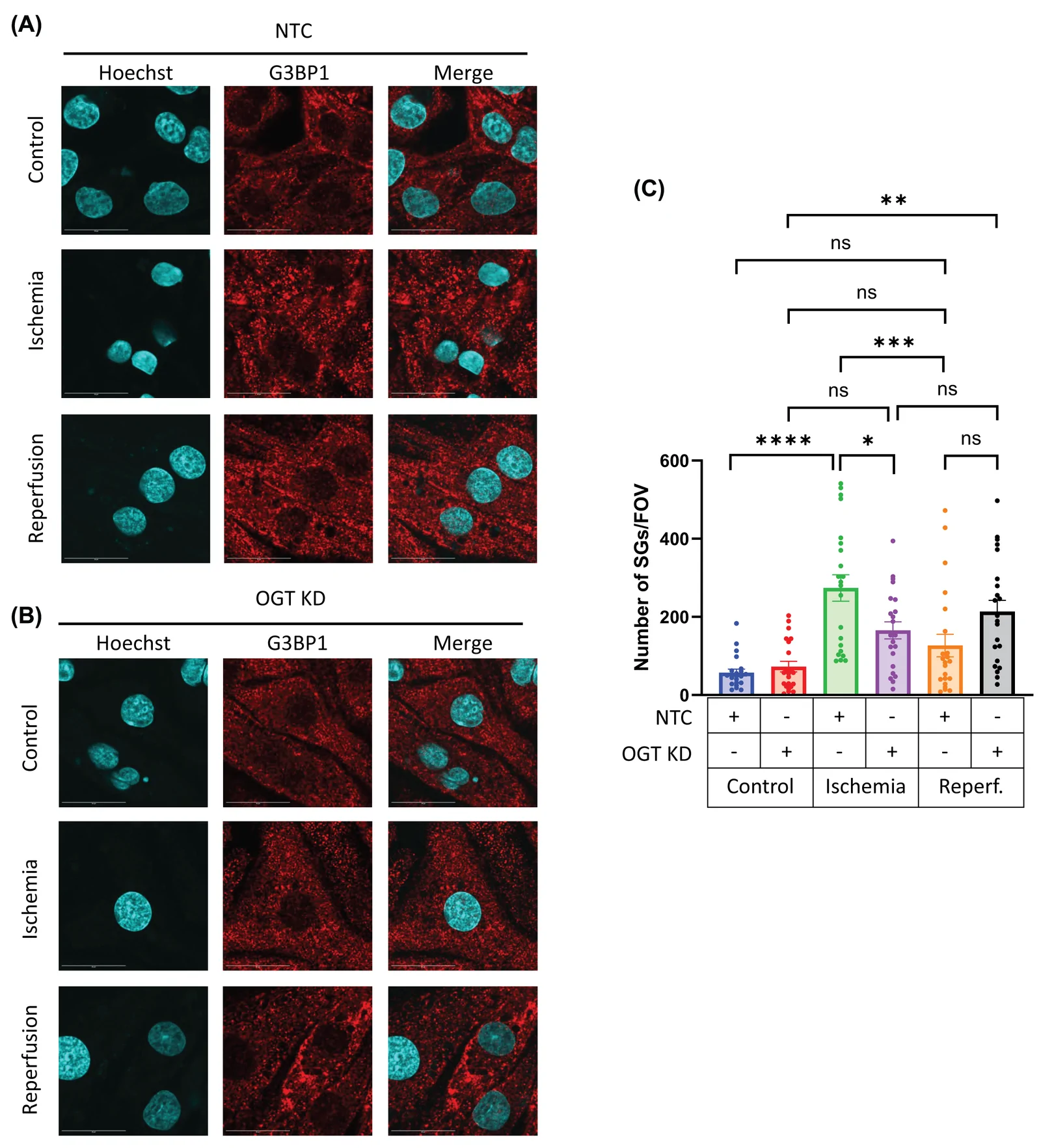
OGT depletion delays stress granule formation in ischemic NRVMs (**A,B**) Representative immunofluorescent images of Hoechst (blue) and G3BP1 (red) staining in NRVMs subjected to simulated I/R (Control, Ischemia, and Reperfusion) following 48 h of siRNA transfection (NTC or OGT-targeting siRNAs, 20 nM; 8 × 10^5^ cells). Images were acquired using an FV3000RS confocal microscope (100× objective). Scale bars: 20 μm. (**C**) Quantification of G3BP1 puncta as SGs/FOV using CellProfiler robust thresholding. *n* = 22 independent image fields from three biological replicates. Data were analyzed using two-way ANOVA followed by Tukey’s post hoc test (****P < 0.0001, ***P < 0.001, **P < 0.01, *P < 0.05; ns not significant).

To investigate the role of OGT in ischemia-induced SG formation, NRVMs were transfected with either non-targeting control (NTC) or OGT-targeting siRNA and subjected to simulated I/R using the coverslip model. G3BP1 immunostaining was employed to monitor SG dynamics (Figure 7A,B). Consistent with our earlier observations, NTC-transfected cells showed a significant increase in SG number during ischemia compared with NTC-control (compare blue and green bars, Figure 7C) and NTC-reperfusion groups (compare green and orange bars, Figure 7C). In contrast, OGT-depleted cells did not display a significant increase in SG number under ischemia relative to their own controls (compare red and purple bars, Figure 7C), while their SG abundance was markedly lower than that of ischemic NTC-transfected cells (compare green and purple bars, Figure 7C, *P* < 0.05). However, there was no statistically significant difference between NTC-transfected and OGT-depleted NRVMs at reperfusion (compare orange and black bars, Figure 7C). It is noteworthy that OGT-depleted reperfused cells appeared to contain more SGs than their control counterparts (compare red and black bars, Figure 7C). Taken together, these data suggest that while OGT-depleted NRVMs subjected to I/R are still capable of forming SGs, their accumulation is attenuated relative to NTC-transfected cells.

### Adaptive stress granules form in ischemic hearts and depend on OGT for efficient assembly

**Figure 8:**
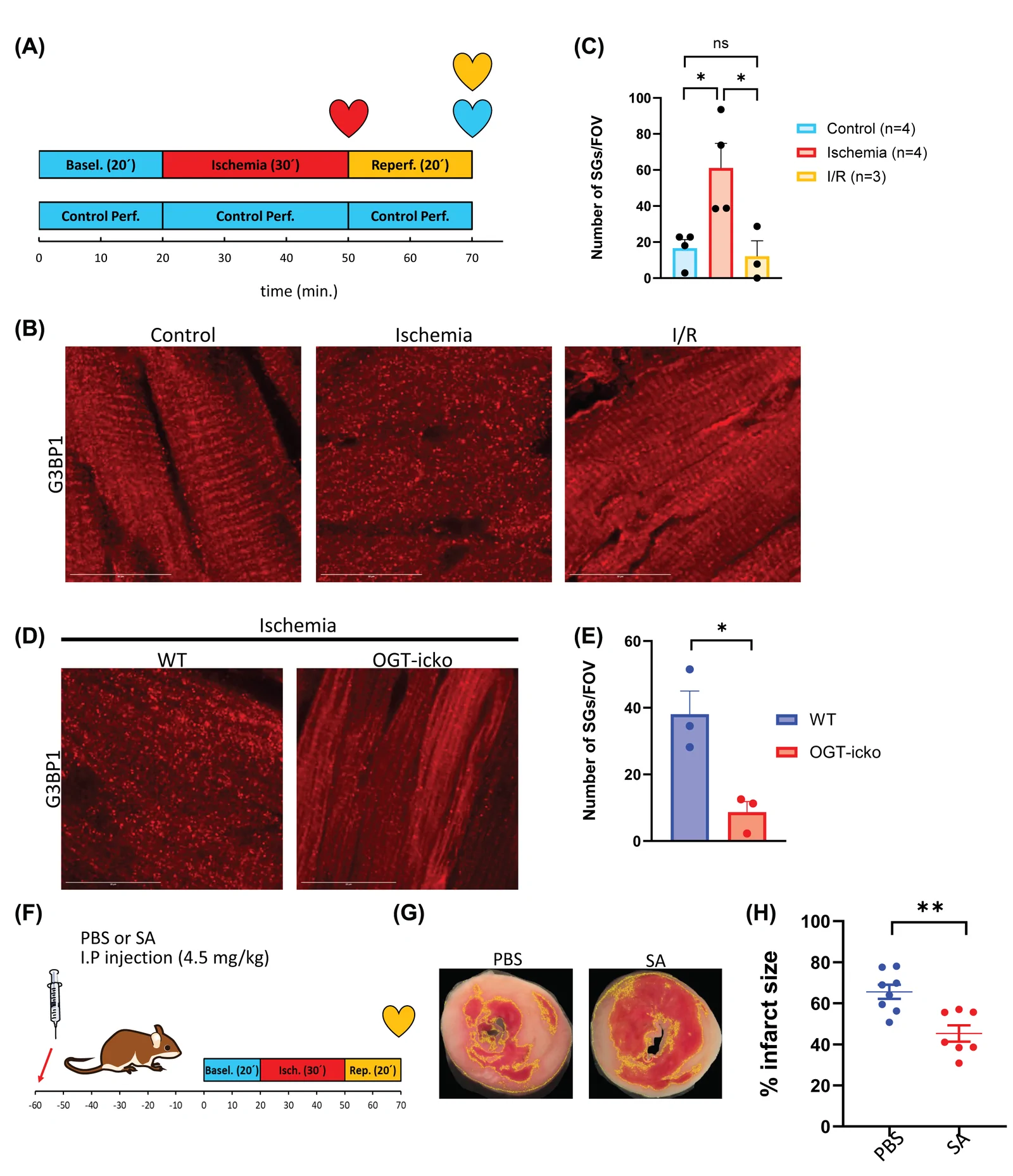
SG formation occurs in mouse heart tissue during ischemia and is reduced by OGT KO (**A**) Diagrams showing the different heart perfusion protocols. Hearts were assigned to three groups: control perfusion (70 min continuous perfusion), ischemia-only (20 min baseline perfusion followed by 30 min index ischemia), and I/R (20 min baseline/30 min index ischemia/20 min reperfusion). (**B**) Mouse hearts from the different experimental protocols were fixed in 4% paraformaldehyde, sectioned, and processed for immunofluorescent staining for G3BP1 to indicate stress granules. Confocal images were acquired on the FV3000RS confocal microscope with a 100× objective. The scale bars are 20 μm. (**C**) Quantification of numbers of SGs/FOV, identified as G3BP1-positive puncta, using CellProfiler with robust thresholding. Data are presented as mean ± SEM from *n* = 3–4 hearts per group. Statistical comparisons were performed by one-way ANOVA with Tukey’s post hoc test (*P < 0.05; ns = not significant). (**D**,**E**) Experiments were performed in inducible, cardiomyocyte-specific OGT knockout (OGT-icko) mice and littermate controls (WT), subjected to the same ischemia-only protocol. (**D**) Representative confocal images showing G3BP1 staining in heart sections. Scale bars are 20 μm. (**E**) Quantification of stress granules as in panel (C). Data are presented as mean ± SEM from *n* = 3 hearts per group. Statistical comparisons were performed using unpaired two-tailed t-test (*P < 0.05). (**F**) Schematic showing the protocol followed to assess the protective effect of sodium arsenite in I/R injury. Mice were injected intraperitoneally with a single bolus dose of sodium arsenite (4.5 mg/kg) or the equivalent volume of PBS, 1 h prior to *ex vivo* perfusion and I/R. (**G**) Representative triphenyl-tetrazolium chloride (TTC)-stained heart slices collected at the end of reperfusion. Viable myocardium is stained red, while infarcted tissue appears pale. Infarct area was quantified using planimetric analysis in Fiji/ImageJ as described in the methods. (**H**) Quantification of infarct size expressed as a percentage of total left ventricular volume, accounting for both the area and the weight of each slice. Infarct size was determined using planimetric analysis in Fiji/ImageJ. Data represent mean ± SEM for PBS (*n* = 8) and sodium arsenite (*n* = 7) groups. Statistical comparisons were performed using an unpaired two-tailed Student’s *t*-test (*P* < 0.01).

Next, we sought to determine whether ischemia-induced SG formation also occurs in intact cardiac tissue. To this end, we employed an *ex vivo* Langendorff-perfused mouse heart model of I/R, which allowed us to perfuse hearts under three experimental protocols (control perfusion, ischemia-only, and I/R, Figure 8A). At the specified time intervals, hearts were collected, fixed, and processed for the histological analysis of SGs, identified as cytoplasmic G3BP1-positive puncta, using confocal microscopy. Quantification across the different groups showed that the ischemic hearts had a significantly higher number of SGs compared with both control-perfused and I/R-perfused hearts (Figure 8B,C), consistent with our experiments on simulated ischemic NRVMs. There were no statistically significant differences between control-perfused and I/R-perfused hearts (Figure 8C).

To verify the role of OGT in SG formation in this context, we employed mice with inducible cardiomyocyte-specific knockout of OGT (OGT-icko) [58]. Reduction of OGT protein abundance in OGT-icko hearts was confirmed by immunoblot analysis of whole-heart homogenates (Supplementary Figure S9A,B). Since our data from simulated I/R conditions in cells and in hearts had demonstrated that ischemic SGs form selectively during ischemia and resolve upon reperfusion, we focused on examining ischemia-only WT and OGT-icko hearts. Immunohistochemical staining for G3BP1 and quantitative analysis of confocal images revealed a robust presence of SGs in ischemic WT hearts that was significantly attenuated in ischemic OGT-icko hearts (Figure 8D,E). These data support that cardiomyocyte OGT is required for the efficient *in vivo* formation of SGs in ischemic hearts, consistent with our findings in OGT-depleted cardiac myocytes subjected to sodium arsenite stress or simulated coverslip ischemia.

Next, we used sodium arsenite, a robust inducer of SG assembly, to assess its potential cardioprotective effect in the context of I/R injury. To that end, mice were injected with a single bolus dose of sodium arsenite (4.5 mg/kg), or an equivalent volume of PBS, 1 h prior to the onset of the I/R protocol (Figure 8F). At the end of reperfusion, hearts were collected, sliced, and stained with TTC to assess the extent of infarct size in the two groups. Remarkably, this analysis showed that sodium arsenite-pretreated hearts had a significantly smaller infarct size compared with PBS controls (Figure 8G,H, *P* < 0.01), consistent with a protective role of sodium arsenite against I/R injury.

### Sodium arsenite-induced stress granules protect H9c2 cells from staurosporine-induced cell death

**Figure 9:**
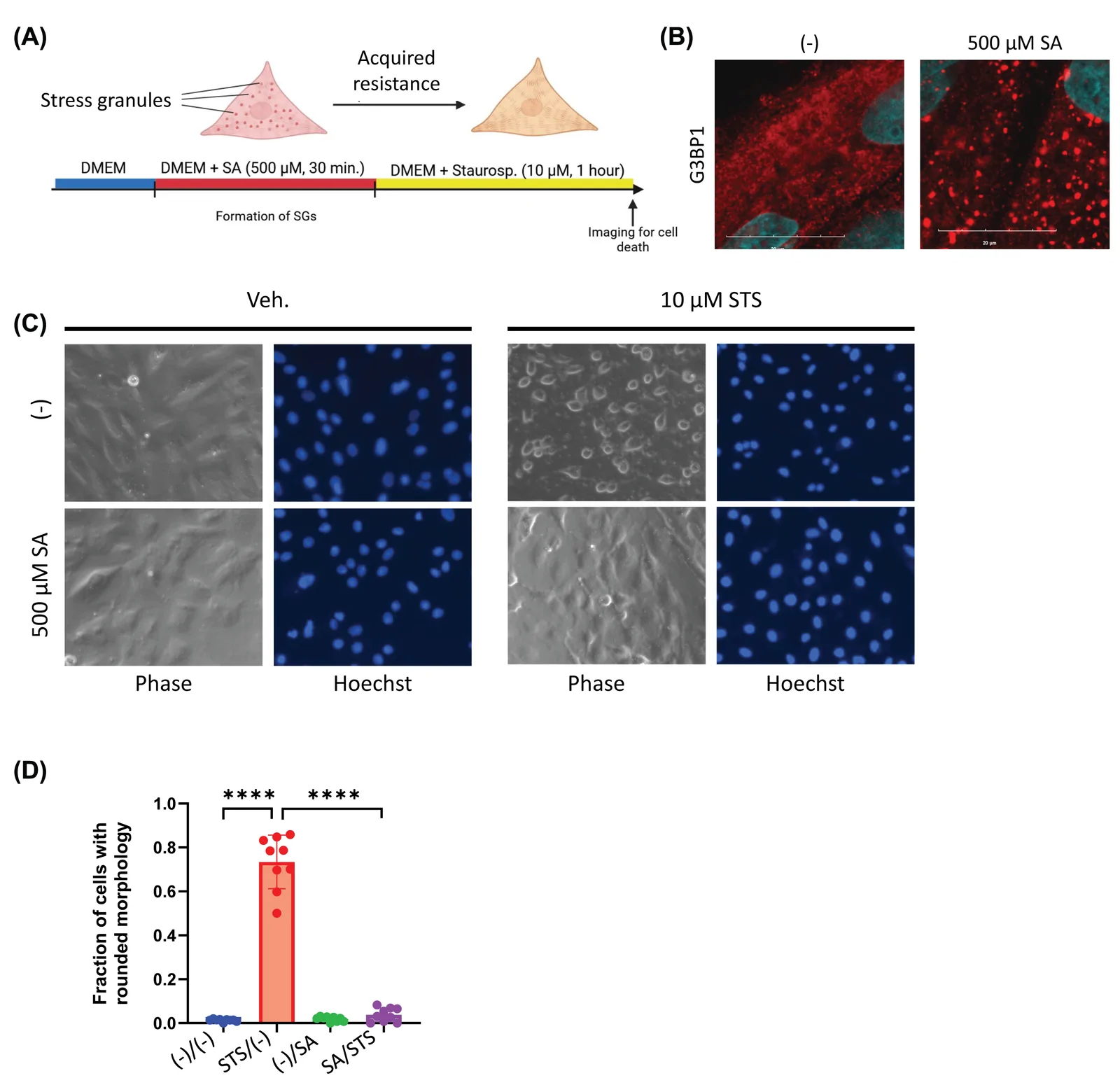
Sodium arsenite-induced stress granule formation protects against staurosporine-mediated cell death in cardiac myocytes (**A**) Schematic illustrating the treatment protocol established to assess the impact of sodium arsenite on cell viability. (**B**) Fluorescent micrographs depicting staining for the stress granule marker, G3BP1, in cells treated or untreated with 500 μM sodium arsenite (SA). Scale bars are 20 μm. (**C**) Representative phase-contrast images of H9c2 cells at the end of the treatment protocol. Cells were pretreated with or without sodium arsenite (500 μM, 30 min) and then challenged with staurosporine (STS, 10 μM) or vehicle (0.1% DMSO) for 1 h. Nuclei were counterstained with Hoechst and images were acquired on an EVOS imaging system equipped with a 20× phase-contrast objective. Cell rounding, assessed by phase-contrast microscopy, was quantified as a morphological marker of acute cell death and normalized to total Hoechst-positive nuclei to provide an index of viability. (**D**) Quantification of cell death expressed as the fraction of rounded up cells normalized to the total number of cells (detected by Hoechst-positive nuclear staining). Data are presented as mean ± SEM. *n* = 9 for all treatment groups. Comparisons across multiple groups resulting from different drug treatments were performed using two-way ANOVA followed by Tukey’ post-hoc test. ****P < 0.0001.

To further corroborate the cytoprotective potential of SGs, we used a cell-based model of cell death. In this approach, cells were transiently treated with sodium arsenite (500 μM, 30 min) before exposure to death-inducing staurosporine (10 μM, 1 h; Figure 9A). As expected, sodium arsenite treatment readily induced the formation of SGs, as shown by immunostaining for G3BP1 (Figure 9B). To monitor cell viability, we assessed cell rounding by phase-contrast microscopy as an early marker of cell death. The number of rounded cells was quantified and normalized to total Hoechst-positive nuclei to provide an index of cell death. As shown with this analysis (Figure 9C,D), we observed a significant increase in the proportion of dead cells caused by staurosporine, and this effect was strongly abolished by pre-treatment with sodium arsenite (Figure 9D, *P* < 0.0001), indicating a protective effect of sodium arsenite–induced SG formation against acute cell death.

**Figure 10:**
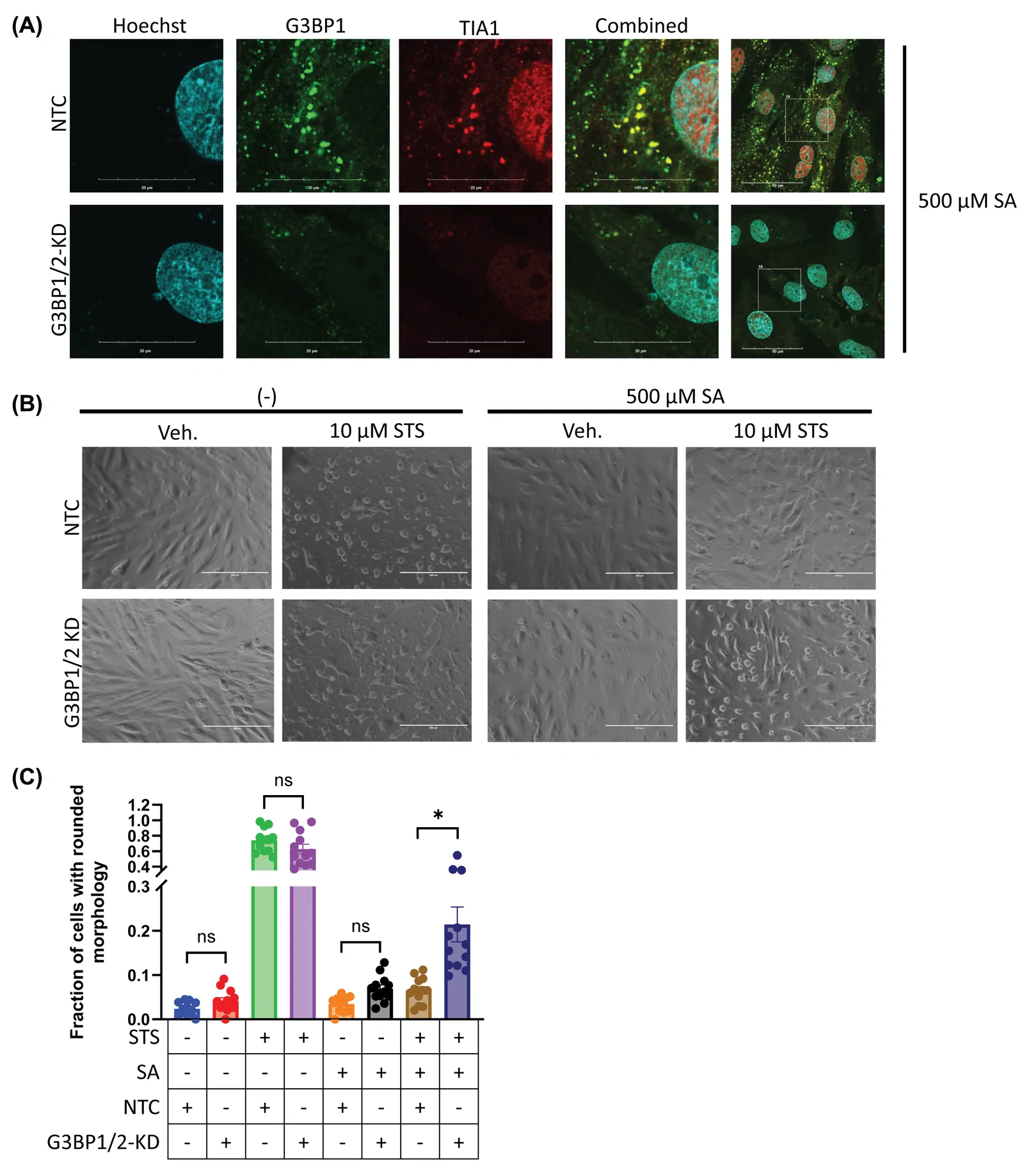
The cytoprotective effect of sodium arsenite is attenuated by genetically depleting the stress granule-forming genes G3BP1/2 (**A**) Representative immunofluorescence showing reduced formation of stress granules following siRNA-mediated knockdown of G3BP1 and G3BP2, compared with NTC. Cells were transfected with siRNA (20 nM, Lipofectamine RNAiMAX) and 48 h later were exposed to sodium arsenite (500 μM, 30 min) to induce the formation of stress granules. After stress exposure, cells were washed and fixed in 4% paraformaldehyde and subsequently processed for immunocytochemistry for stress granule markers G3BP1 and TIA1. Nuclei were counterstained with Hoechst. Scale bar: 20 μm. (**B**) Representative phase-contrast images of cells subjected to sodium arsenite pretreatment (500 μM, 30 min) followed by either staurosporine (STS) challenge (10 μM, 1 h) or vehicle control (0.1% DMSO). Cell death was assessed as morphological rounding, normalized to Hoechst-positive nuclei. Scale bar: 20 μm. (**C**) Quantification of cells with rounded morphology in NTC or G3BP1/2-KD cells and across the different treatment conditions. Cells were seeded in 8-chamber slides and transfected with NTC or G3BP1/2 siRNAs (20 nM each). Forty-eight hours later, cells were exposed to staurosporine challenge with or without prior sodium arsenite pretreatment. Comparisons across multiple groups resulting from genetic knockdown and drug treatments were performed using two-way ANOVA with Tukey’s post-hoc test. *P < 0.05, ns: not significant, *n* = 9 replicates.

To further investigate whether the cytoprotective effect of sodium arsenite was mediated by SG assembly, we genetically targeted the SG orchestrators G3BP1 and its close homolog G3BP2 [33]. Effective reduction of G3BP1 and G3BP2 protein abundance by siRNA-mediated knockdown was confirmed by immunoblot analysis (Supplementary Figure S10). Next, cells were transfected with siRNAs targeting G3BP1 and G3BP2 or with NTC and exposed to sodium arsenite treatment for 30 min. Immunofluorescence analysis was then performed to assess the efficiency of SG formation in G3BP1/2-targeted cells. Indeed, the knockdown approach significantly impaired the formation of SGs, as assessed using G3BP1 immunostaining (Figure 10A). Further confirmation was also provided by staining for the independent SG marker, TIA1. This staining showed a defect for TIA1 to coalesce into distinct SGs upon sodium arsenite treatment (Figure 10A), consistent with the deficit in SG formation caused by G3BP1/2-knockdown. Next, we examined the impact of G3BP1/2 depletion on sodium arsenite-induced cytoprotection. While G3BP1/2 depletion did not affect basal or staurosporine-only-induced cell death, it caused a significant increase in cell death in cells pretreated with sodium arsenite and exposed to staurosporine (compare brown and lavender blue, Figure 10B,C, *P* < 0.05). Collectively, these findings support the conclusion that SG assembly downstream of sodium arsenite treatment contributes significantly to the cytoprotective effect against staurosporine treatment.

**Figure 11:**
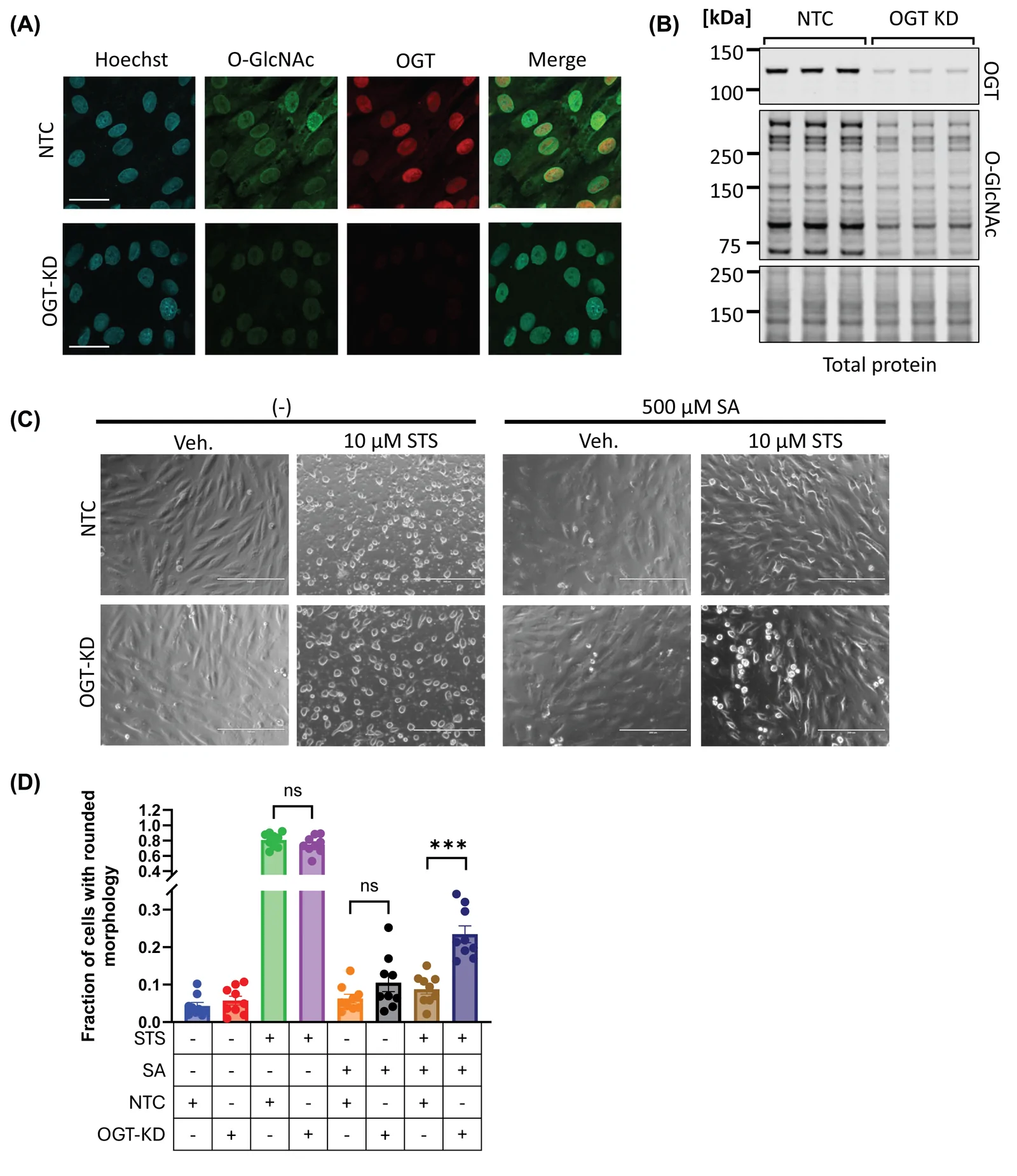
OGT depletion attenuates sodium arsenite-mediated protection against staurosporine-induced cell death (**A**) Representative immunofluorescence showing reduced O-GlcNAc (green) and OGT (red) signal following siRNA-mediated knockdown of OGT, compared with NTC. Cells were transfected with siRNA (20 nM) using Lipofectamine RNAiMAX and fixed 48 h after transfection for immunocytochemistry. Nuclei were counterstained with Hoechst. (**B**) Western blot analysis in NTC siRNA- or OGT siRNA-transfected cells to assess for OGT protein levels and overall changes in O-GlcNAcylation. Similar to A, cells were transfected with 20 nM siRNAs and processed for analysis 48 h later. (**C**) Representative phase-contrast images of cells subjected to sodium arsenite pretreatment (500 μM, 30 min) and then exposed to staurosporine (STS) challenge (10 μM, 1 h) followed by imaging on the EVOS system. Cell death was assessed as morphological rounding normalized to Hoechst-positive nuclei. Scale bar: 20 μm. (**D**) Quantification of cell death fraction in NTC or OGT-KD cells and across the different treatment conditions. Cells seeded in 8-chamber slides were transfected with NTC or OGT siRNAs and, 48 h later, were exposed to STS challenge with or without prior sodium arsenite pretreatment. Statistical analysis was performed using two-way ANOVA with Tukey’s post hoc test ***P < 0.001, ns: not significant, *n* = 9 replicates.

To address the importance of OGT and O-GlcNAcylation in the context of SG cytoprotection, we targeted OGT using siRNA. Immunofluorescence analysis demonstrated a marked reduction in OGT signal, both in the cytoplasm and the nucleus of cells, consistent with effective knockdown (Figure 11A). Consistently, western blot analysis showed substantially lower OGT levels in OGT siRNA-transfected cells versus NTC-transfected cells, and the O-GlcNAcylation levels were visibly decreased in OGT-depleted cells (Figure 11B). Next, we assessed the propensity of OGT-depleted cells to undergo cell death at baseline and in staurosporine-treated conditions with or without prior treatment with sodium arsenite (Figure 11C). OGT depletion did not affect cell death at baseline or in response to staurosporine or sodium arsenite alone. By contrast, sodium arsenite pretreatment failed to fully protect OGT-depleted cells, which showed significantly greater cell death upon staurosporine challenge than NTC controls (compare brown and lavender blue, Figure 11D, *P* < .001). These results indicate that OGT is required for the full protective effect of sodium arsenite pretreatment and SG formation, indicating a novel link between OGT and granule-mediated cytoprotection.

## Discussion

Stress granules are increasingly recognized as central regulators of cellular stress responses and are implicated in diverse diseases, including neurodegeneration and cancer [59–62], yet their regulation and role in the context of cardiac injury remain largely unexplored. Here, we demonstrate that O-GlcNAcylation is an important determinant of stress granule formation in cardiac cells. Using complementary time-lapse imaging approaches and genetic approaches, we establish that OGT activity is necessary for efficient stress granule assembly in cardiac myocytes. Using protein enrichment and O-GlcNAc site mapping on the stress granule orchestrator G3BP1, we identified T268 as an O-GlcNAcylated residue with functional significance. Importantly, we show that stress granules form in cardiomyocytes under ischemic conditions and dissipate upon reperfusion, and that OGT plays an important role in stress granule formation in the ischemic heart. Finally, we found that pharmacologic induction of stress granules in cultured cardiomyocytes confers resistance to acute cell death and that pre-emptive induction of stress granules *in vivo* by sodium arsenite protects hearts against I/R injury. Collectively, our findings identify OGT and O-GlcNAcylation as a central regulatory modality coordinating stress granule assembly and promoting cardiomyocyte adaptation and survival during ischemic stress.

Our data establish OGT activity as a critical mediator of stress granule formation in cardiac cells, consistent with previous work showing that OGT depletion delays arsenite-induced stress granule formation in other cell types [37,63]. Interestingly, our pharmacological or genetic inhibition of OGA did not accelerate stress granule formation. These findings are consistent with earlier experiments showing that shRNA-mediated depletion of OGA/MGEA5 did not affect arsenite-induced stress granule assembly in cells [37]. One possible explanation for the lack of effect by OGA inhibition is that in our experimental conditions arsenite elicits a near-maximal stress granule response on its own, leaving little room for additional potentiation to be observed upon OGA inhibition. Alternatively, OGA may exert a greater influence on stress granule disassembly or clearance rather than formation, a role that would not be captured within the time frame of the stress granule formation assays used here. Intriguingly, our data show that OGA, but not OGT, is recruited to stress granules, suggesting that OGA may be required for later remodeling steps rather than in the initial granule nucleation events. Moreover, the absence of OGT from stress granules aligns with a model where proteins already O-GlcNAcylated are preferentially sequestered, rather than being modified after being recruited into the stress granules. Consistent with this model, sodium arsenite treatment did not produce an apparent increase in the total O-GlcNAc signal detected on G3BP1 over the tested time window, suggesting that acute stress granule assembly does not require a gross increase in G3BP1 O-GlcNAcylation.

Following the initial observation linking the stress granule inducer sodium arsenite with O-GlcNAcylation [14], multiple stress granule constituents across diverse functional classes have been reported as O-GlcNAc targets. These include ribosomal subunits [37], translation-initiation factors such as eIF4G1 [64], and the core scaffold Caprin1 [38]. In addition, DEAD-box (DDX) RNA helicases feature frequently both as stress granule components [65,66] and as O-GlcNAc targets (e.g. DDX3X) [67,68]. Similarly, heterogeneous nuclear ribonucleoproteins (hnRNPs) are recurrent stress-granule constituents [69,70] and carry O-GlcNAc modifications (e.g. hnRNPA1) [71]. However, in most of these studies, O-GlcNAcylation is not functionally linked to the regulation of stress granule formation or dynamics. In this regard, our demonstration that the core nucleator G3BP1 is O-GlcNAcylated provides key evidence supporting O-GlcNAcylation as an active regulator of stress granule formation.

G3BP1 is a modular RNA-binding protein comprising an NTF2L domain, an acidic middle region that contains a proline-rich domain (PxxP, amino acids 221–339), and a C-terminal region that contains the RRM/RG-rich domains [32,33,43,72]. Our work identified T268 as an O-GlcNAcylation site with functional significance. Notably, although T268 contributes to stress granule assembly, mutation of this site did not eliminate the total O-GlcNAc signal detected at the G3BP1 band, suggesting that additional O-GlcNAc sites on G3BP1 remain to be identified. T268 lies within the disordered acidic region and within the boundaries of the PxxP domain. The PxxP domain of G3BP1 constitutes a canonical SH3-binding interface [44] and has been suggested to have important roles in the antiviral activity of G3BP1, but also in the initial nucleation events of stress granules [73,74]. Given PxxP’s role in protein–protein interactions, it is possible that the presence of O-GlcNAc in that region could sterically weaken the binding of putative SH3-containing proteins, favoring condensation and stress granule nucleation. Moreover, the acidic region forms intramolecular contacts with the C-terminal RGG/RG-rich tail that masks the RRM and limits RNA engagement during stress-granule assembly [32,33]. In this regard, it is possible that the presence of O-GlcNAc at T268 weakens these intramolecular contacts, effectively relieving autoinhibition, favoring G3BP1:RNA interactions and promoting stress granule nucleation.

Stress granule formation is a multi-step process that frequently involves liquid–liquid phase separation, as untranslated mRNPs and associated proteins coalesce into droplet-like clusters [31,75,76]. Evidence from different experimental systems indicates that O-GlcNAcylation on individual proteins can either promote or dampen their phase separation. For example, O-GlcNAcylation on the RNA-binding protein, EWS, reduces its phase separation *in vitro* and enhances the liquid-like nature of EWS condensates [77]. Consistently, independent work correlated EWS O-GlcNAcylation with reduced partitioning into cytoplasmic granules during hyperosmotic stress [78]. Furthermore, NMR work on CAPRIN1 indicates that O-GlcNAcylation on S644/S649 lowers its phase-separation propensity by weakening key intermolecular interactions [79]. In another experimental system, more relevant to synaptic transmission, O-GlcNAcylation of SynGAP at T1306 blocks its interaction with PSD-95 and suppresses phase separation of the SynGAP/PSD-95 assembly [80]. Finally, a study investigating the biological functions of *N*^6^-methyladenosine (m^6^A)-RNA reader proteins showed that YTHDF1/3 undergo O-GlcNAcylation and that this dampens their condensation into stress granules [81]. Additionally, O-GlcNAcylation of YTHDF1/3 is suggested to be important for their dynamic movement in and out of stress granules and favoring a more efficient disassembly of the stress granules [81]. In an independent study, however, O-GlcNAcylation on YTHDF3 appears to promote the formation of YTHDF-containing stress granules [82]. The same was also observed for another protein, NUFIP2. Moreover, OGT inhibition with OSMI in that setting was inhibitory for condensate formation [82]. Taken together, these findings suggest a more nuanced role of O-GlcNAcylation’s effects that appear to be protein- or even context-dependent. In our experimental systems (arsenite-induced stress granule formation, simulated coverslip I/R injury, and whole heart I/R injury) global O-GlcNAcylation suppression delays stress granule assembly, consistent with the initial work placing O-GlcNAcylation upstream of stress granule regulation [37]. Moreover, our mutational analysis further implicates O-GlcNAcylation on G3BP1 as a positive determinant of stress granule formation.

In primary rat cortical neurons or PC12 cells subjected to oxygen-glucose deprivation/reperfusion, TIA1/G3BP1-positive stress granules were shown to form during the 6 h of deprivation and then progressively decreased upon reperfusion [83,84]. TIA1/G3BP1-containing stress granules are also detected in the cerebral cortex in a model of focal cerebral I/R, and their formation is associated with ameliorated injury [84]. These findings are consistent with earlier studies of global brain ischemia documenting translational arrest and formation of TIA1-containing stress granules rise early in reperfusion (∼10 min) and decline by ∼90 min [85]. Furthermore, brain regions that assemble stress granules promptly at reperfusion and resolve them rapidly (i.e. Cornu Ammonis, CA3) are relatively injury-resistant, whereas regions with delayed SG dynamics (e.g. CA1) are more vulnerable and later show convergence with ubiquitin-positive protein aggregates [85,86]. The increased vulnerability in CA1 was also associated with a non-canonical pathway for stress granule formation [54]. Stress granules were also detected in the context of renal I/R [87]. Complementing these I/R observations, metabolic inhibition (combining glycolytic blockade with ATP depletion in neuronal cells) stimulates the formation of stress granules [88]. Moreover, efficient recovery of ATP synthesis is necessary for the effective clearance of stress granules [88]. In the heart, within 1 h of coronary occlusion, eIF4G redistributes into TIAR-positive RNP granules [89], consistent with the formation of stress granule-like structures in the ischemic myocardium. Taken together, these findings are consistent with our data both in cardiomyocytes exposed to simulated I/R injury and in I/R-injured hearts, demonstrating that stress granules assemble transiently over the course of ischemia and then resolve upon reperfusion.

Across different experimental systems, the rapid assembly of stress granules is cytoprotective. For example, blocking stress granule assembly by oxidizing TIA1 increases apoptosis, and depleting the core nucleators G3BP1/2 heightens stress-induced cell death [90,91]. Mechanistically, assembled stress granules are suggested to be cytoprotective by sequestering executioner caspases and other death-inducing factors [50,91–94]. Accordingly, stress granules have been shown to be cytoprotective in the context of viral infection, proteotoxic stress, and lysosomal damage [95–97]. In this work, we developed an on/off strategy that first exposes cells to sodium arsenite to induce stress granules, followed by arsenite wash-off and challenge by staurosporine, which robustly activates intrinsic apoptotic pathways [98,99], yet it does not elicit stress granules [91,100]. In this setup, arsenite priming protected against staurosporine-induced killing, and critically, this benefit was significantly attenuated by G3BP1/2 depletion. While arsenite can trigger broader stress responses, our data indicate that stress granule assembly is required for the full protective effect observed in this setting and align with the notion that acute, transient stress granule formation is an important survival mechanism.

In addition to a cytoprotective effect in transiently exposed cells, our work showed that acutely injecting sodium arsenite to mice 1 h prior to I/R injury has a significant cardioprotective effect, evident very early at reperfusion. These results are consistent with multiple preclinical studies showing that acute, single-dose arsenite can act as a chemical preconditioning stimulus. For example, one study reported that arsenite bolus (4.5 mg/kg) given 30 min before coronary occlusion reduced infarct size, although this effect was associated primarily with effects on mitochondrial respiration, and inflammation and was observed late in reperfusion (24 h) [101]. Beyond the heart, studies on kidney and liver demonstrated that arsenite pretreatment (6 or 8 mg/kg) limits apoptosis and tissue injury markers after I/R, and in these studies the effects were associated with the induction of Hsp70 and were again observed mid-to-late in reperfusion (i.e. 6 or 24 h) [102,103]. However, it should be noted that outside a narrow dose and time window, prolonged arsenite administration at the micromolar level can induce cardiac, smooth muscle, or endothelial cell death [104–106]. Moreover, low-dose exposure to arsenite (at submicromolar levels) over the period of weeks (typically from 4 to 22) is known to induce or exacerbate adverse remodeling in preclinical cardiac models [107–110], and chronic exposure to arsenite has been viewed as an environmental pollutant contributing to cardiovascular disease [111]. Taken together, arsenite’s effects on the heart are highly dependent on dose and exposure duration. Our data have identified an acute, pre-ischemic window in which transient arsenite administration confers cardioprotection, and we attribute this response to a stress-granule–linked adaptation in the acutely ischemic heart.

Prior work shows that targeting OGT in cardiomyocytes sensitizes the heart to injury and adverse remodeling [24], while acute augmentation of O-GlcNAc is protective in stressed cardiac myocytes [27,112] and in I/R-injured hearts [20,22,23]. Similar directionality holds in the brain, where moderate O-GlcNAc elevation lessens stroke injury and neuron-specific OGT deletion aggravates regional cerebral I/R injury outcomes [113,114]. While the cardio- and neuroprotective effects of OGT in these contexts are likely to be multifaceted, our data identify a specific requirement for OGT in efficient assembly of G3BP1-positive stress granules under ischemic stress. These findings in isolated cardiomyocytes were also recapitulated in perfused OGT-deficient hearts exposed to ischemic stress, providing genetic *in vivo* evidence that OGT coordinates stress granule assembly in the ischemic myocardium. Moreover, in our arsenite/staurosporine on/off experiment, arsenite priming reduced staurosporine cytotoxicity, and this benefit was blunted by OGT depletion, supporting a contributory role for OGT in stress granule-linked cytoprotection.

Together, these data show that O-GlcNAcylation promotes timely assembly of G3BP1-defined stress granules in cardiomyocytes exposed to arsenite or ischemic stress. We map a functionally important O-GlcNAc site on G3BP1 and propose that pre-existing O-GlcNAcylation primes stress granule proteins to coalesce in a timely manner. Pre-emptive formation of stress granules in cardiac myocytes is cytoprotective as it preserves proteostasis and suppresses pro-death signaling, particularly during the vulnerable early reperfusion window. These findings define a novel mechanistic framework connecting O-GlcNAcylation, G3BP1, and stress granules in stressed cardiac myocytes and ischemic hearts, opening opportunities for new therapeutic approaches in ischemic and related cardiac conditions.

## Experimental procedures

### Materials

A detailed list of key reagents, including their identifiers, vendors, and catalog numbers, is provided in Supplementary Table S3. Additional information on other reagents can also be found in the description of methods in the text below. Procedures involving animals were performed at the Johns Hopkins University School of Medicine and were approved by the Institutional Animal Care and Use Committee (IACUC) in accordance with institutional and NIH guidelines. All mice were maintained on a C57BL/6J background and housed under standard laboratory conditions with free access to food and water. The generation of the inducible cardiomyocyte-specific OGT knockout mice was previously described [58]. *Ogt*-floxed mice carrying the *MerCreMer-Myh6-Cre* transgene or their cre-negative littermates were treated with tamoxifen by intraperitoneal injection for four consecutive days (20 mg/kg/day). Langendorff perfusion experiments and heart collection were performed 10 days after the final tamoxifen injection. Certain schematics were generated using BioRender.

### Plasmids and adenoviral constructs

A recombinant adenoviral vector expressing enhanced green fluorescent protein (EGFP, codon optimized) fused to the N-terminus of human G3BP1 (RefSeq: NM_005754.3) was custom-designed by VectorBuilder (Chicago, IL, U.S.A.). The construct included a Kozak consensus sequence (GCCACCATGG) harboring the start codon to enhance translation initiation. The coding sequence was inserted into a deleted adenovirus serotype 5 (ΔAd5) viral backbone under the control of the CBH promoter (hybrid promoter composed of the cytomegalovirus (CMV) enhancer and chicken β-actin promoter with a hybrid intron). A second adenovirus carrying a threonine-to-alanine point mutation at residue 268 (T268A) in G3BP1 was generated using the same construct design and backbone.

A separate recombinant adenoviral vector used for bioorthogonal metabolic-labeling experiments was custom-designed by VectorBuilder to express human UAP1-F383G containing N-terminal Myc and FLAG epitope tags. The F383G point substitution was introduced into the human UAP1 open reading frame by site-directed mutagenesis. The UAP1-F383G expression cassette was inserted into a ΔAd5 viral backbone under the control of a cardiac troponin T promoter and included a ribosome-skipping 2A sequence followed by EGFP to monitor successful transduction and transgene expression.

Adenoviruses were packaged in HEK293 producer cells, amplified to high titer (∼1 × 10^12^ viral particles/ml), purified by two sequential cesium chloride gradients, dialyzed, and titrated by UV spectrophotometry and confirmed by plaque formation assay. Cells were transduced with each virus at a multiplicity of infection (MOI) of 50, 24 h prior to experiments, unless otherwise specified.

### Cell culture of H9c2 cells, siRNA transfection, and adenoviral transduction

H9c2 rat cardiomyoblasts (ATCC) were cultured in DMEM (4.5 g/l glucose) supplemented with 10% (v/v) fetal bovine serum and 100 U/ml penicillin–streptomycin (both from Gibco), along with 2 mM GlutaMAX, MEM non-essential amino acids, and 10 mM HEPES (all from Invitrogen). For gene knockdown, cells were transfected with Dicer-substrate siRNA (DsiRNA) using Lipofectamine RNAiMAX (Thermo Fisher Scientific) 48 h prior to experimentation. Transfection complexes were formed at room temperature for 15 min according to the manufacturer’s protocol and added at a final ratio of 40 pmol per 1 × 10^6^ cells. A non-targeting DsiRNA (IDT) was used as control. The sequences of all DsiRNAs used are provided in Supplementary Table S3.

To monitor rates of stress granule formation, cells were transduced with adenovirus encoding EGFP–G3BP1 (MOI = 50) 24 h prior to confocal imaging. Cells were treated with the indicated pharmacological inhibitors for durations specified in the figure legends and main text.

### Live cell imaging, data acquisition, and analysis

For stress granule induction, 500 μM sodium arsenite was added directly to the culture medium, and cells were imaged over the subsequent 20 min at 1 min intervals. Imaging was performed on an Olympus FV3000 scanning confocal microscope equipped with a 100×/1.45 NA oil-immersion objective at 37°C and 5% CO_2_. Nuclei and G3BP1 signals were visualized using 405 nm and 488 nm lasers, respectively, in the presence of NucBlue.

For data analysis, .oir images were uploaded into a Fiji-run custom-made macro to convert composite images into individual .tiff single-color channels (blue, green, and red). The images were then uploaded into CellProfiler (version 4.2.1.; https://cellprofiler.org/). The CellProfiler pipeline extracts nuclei from the blue channel and uses those as primary objects. Next, using nuclei as primary objects, the pipeline identifies the cells from the G3BP1 signal using the ‘Propagation’ method. Stress-granule puncta were segmented and quantified in CellProfiler using the ‘RobustBackground’ thresholding feature and summarized as total counts per FOV at 100× magnification under identical acquisition settings. FOVs were chosen from regions with high cell coverage. The main output parameter of the pipeline used for quantification of stress granule formation was ‘Count Stress Granules.’

### Cell culture of NRVM cardiomyocytes, siRNA transfection

NRVMs were isolated from P0-P1 rats of either sex using enzymatic dissociation, according to a standard methodology as previously described [115,116]. Following a pre-plating step to remove fibroblasts and other non-myocytes, the cardiomyocytes (suspensions at 0.5 × 10^6^ cells/ml) were seeded for experiments on culture plates pre-coated with 10 μg/ml fibronectin (Sigma–Aldrich) and incubated at 37°C, 5% CO_2_ for 24 h. Typical seeding densities were 1.0 × 10^6^ cells per well in a six-well plate for western blot experiments, or 0.8 × 10^6^ cells per chamber in glass-bottom cell culture dishes (NEST) for confocal microscopy. The following day, cells were switched to serum-free DMEM (Corning) supplemented with insulin–transferrin–selenium (ITS; Gibco) and 10 mM HEPES (Invitrogen). For knockdown experiments, cells were transfected with a Lipofectamine/siRNA mix 48 h prior to treatment.

### NRVM model of simulated I/R injury by glass coverslip application

Prior to the experiment, the culture medium was replaced with fresh Tyrode’s buffer (130 mM NaCl, 5 mM KCl, 1 mM MgCl_2_, 10 mM NaHEPES, 1 mM CaCl_2_, and 5 mM glucose). Simulated ischemic injury was induced by placing a glass coverslip over the cells for 60 min to restrict oxygen and nutrient exchange, as previously described [57,117]. Where indicated, simulated reperfusion was achieved by removing the coverslip and incubating the cells for an additional 60 min in Tyrode’s buffer. Control cells were incubated in Tyrode’s buffer for 120 min without coverslip placement.

### Immunocytochemistry staining and confocal imaging of NRVMs subject to simulated I/R injury

Following coverslip applications, NRVMs from the different treatment groups (Control, Ischemia, and Reperfusion) were washed with 37°C prewarmed PBS and then fixed with prewarmed 4% paraformaldehyde for 15 min. The fixative was then washed off with PBS and cells were permeabilized with 0.1% Triton X-100 for 5 min at room temperature. This was followed by PBS washes and blocking overnight at 4°C with 2.5% BSA and 2.5% normal goat serum (NGS, Cell Signaling Technology, catalog number: 5425) in 0.1% tween-20 PBS (PBS-T). Primary antibodies against O-GlcNAc (200:1, RL2, host mouse) or G3BP1 (1000:1, Cat. No. 13057-2-AP, host rabbit) were diluted in blocking solution (2.5% BSA, 2.5% normal goat serum, 0.1% Tween-20 in PBS) and incubated with samples at 4°C with gentle shaking overnight.

For independent antibody validation, cells were stained with mouse anti-G3BP1 in combination with a rabbit anti-O-GlcNAc multiMAb antibody mix (Cell Signaling Technology, Cat. No. 82332). This reagent consists of individual rabbit monoclonal antibody clones combined in optimized ratios and selected for motif recognition and assay performance to provide broad detection of O-GlcNAc-modified proteins. After washes with PBS-T (3×), species-appropriate Alexa Fluor 488- or Alexa Fluor 546-conjugated secondary antibodies were applied for 1 h at room temperature in blocking solution. Nuclei were counterstained with Hoechst 33342 (5000:1 in PBS, 5 min). Imaging was carried out with the Olympus FV3000RS confocal microscope using the 100× objective and 405, 488, and 561 nm lasers to excite Hoechst, Alexa 488- and Alexa 546-conjugated immunocomplexes, respectively. Laser power and gain were kept constant across imaging sessions, and four fields of view were captured per plate.

### Immunocytochemistry and image acquisition

Cells plated on ibidi chamber slides (4-well or 8-well, Supplementary Table S3) were treated with or without sodium arsenite (500 μM, 30 min), washed with 37°C prewarmed PBS (3×), and fixed with prewarmed 4% paraformaldehyde in PBS for 15 min. The fixative was removed by washing with PBS (3×), and cells were permeabilized with 0.1% Triton X-100 in PBS for 5 min at room temperature. Blocking was performed with 2.5% BSA, 2.5% normal goat serum, and 0.1% Tween-20 in PBS for 1 h at room temperature. For SG immunofluorescence in NRVMs, cells were stained with one of the following SG markers: G3BP1, eIF4G1, PABPC1, or TIA1 (all raised in rabbit and used at a dilution of 200:1), in combination with the RL2 anti-O-GlcNAc antibody (raised in mouse and used at a dilution of 200:1). For RL2 competition experiments, the RL2 antibody was preincubated with 500 mM free GlcNAc before application to fixed cells, followed by the standard immunostaining procedure. For independent antibody validation, cells were stained with anti-G3BP1 raised in mouse in combination with a rabbit anti-O-GlcNAc multiMAb, as indicated in the corresponding figure legend. Primary antibodies were added overnight at 4°C with gentle shaking. After washes with PBS-T (3×), species-appropriate Alexa Fluor 488- or Alexa Fluor 546-conjugated secondary antibodies were applied for 1 h at room temperature in blocking solution. Additional antibody combinations and dilutions are provided in the corresponding figure legends. Nuclei were counterstained with Hoechst 33342 (5000:1 in PBS, 5 min). Images were acquired on an Olympus FV3000RS confocal microscope using a 100×/1.35 NA silicone oil immersion objective (UPLSAPO100XS).

### Colocalization analysis of OGT and OGA in stress granules

For colocalization analysis of OGT or OGA with SGs, cells were processed for immunocytochemistry as described above. Samples were incubated overnight at 4°C with anti-OGT (AL24, raised in rabbit; 200:1) or anti-OGA (Cat. No. 14711-1, raised in rabbit; 200:1), together with anti-G3BP1 (Cat. No. 66486-1-Ig, raised in mouse; 1000:1). Fluorescence detection was performed using Alexa Fluor 488- and Alexa Fluor 546-conjugated secondary antibodies, and nuclei were counterstained with Hoechst 33342 as described above.

Co-localization between G3BP1 and OGT or G3BP1 and OGA within granules was quantified in CellProfiler using the MeasureColocalization module. Stress granule masks were generated from the G3BP1 channel and segmented with IdentifyPrimaryObjects using two-class global Otsu thresholding (threshold smoothing scale = 1; correction factor = 3; bounds = 0.005–1.0), typical diameter 5–100 pixels, and shape-based declumping (smoothing filter = 10 pixels; minimum peak distance = 7 pixels; border objects discarded). Pearson’s correlation coefficients were computed using MeasureColocalization for G3BP1↔OGT and G3BP1↔OGA pixel intensities restricted to the stress granule masks.

### Langendorff I/R injury

The procedure to perfuse isolated mouse hearts and subject them to I/R injury was performed as previously described [57]. Briefly, a controlled digital peristaltic pump (Reglo, Ismatec) was used to deliver the buffer at a constant flow through Tygon tubing (Cole-Parmer). The freshly made perfusion buffer (Krebs-Henseleit, consisting of 118 mM NaCl, 4.7 mM KCl, 25 mM NaHCO_3_, 1.2 mM KH_2_PO_4_, 10 mM glucose, 1.2 mM MgSO_4_, and 2 mM CaCl_2_, pH 7.4) was vacuum-filtered and loaded onto the perfusion apparatus, where it was continuously bubbled with a mixture of 95% CO_2_ and 5% O_2_ and warmed at 37°C. The temperature in the perfusate right above the heart was monitored (NBD TC2 temperature controller). One hour prior to heart excision, mice were injected intraperitoneally with a single bolus dose of sodium arsenite (4.5 mg/kg) or the equivalent volume of PBS. After 45 min, the mice were injected intraperitoneally with heparin (200 U/g body weight, Sagent) and 15 min later, they were anesthetized with 2,2,2-tribromoethanol (0.2 mg/g body weight). After deep anesthesia the hearts were quickly excised, weighed, cannulated through the aorta, and mounted onto the perfusion rig. Perfusions were performed at the constant pressure mode corresponding to 70 mmHg. The heart was immersed in 37°C perfusion buffer warmed in water-jacketed tissue bath (Radnoti) and allowed to beat with its intrinsic rate without pacing. After an initial phase of normoxic perfusion for 20 min, the hearts underwent global no-flow (ischemia) for 30 min followed by 20 min of reperfusion.

At the end of reperfusion, the hearts were arrested in diastole by switching to KH supplemented with KCl (1% w/v) and then perfused with TTC (1% w/v in KH, 37°C) for 5 min until viable tissue developed a uniform red coloration. Following TTC staining, the hearts were removed from the rig, rinsed in PBS, and sliced into short-axis slices (1–2 mm thickness) from apex to base. Slices were placed between two coverslips and photographed at both sides using a stereoscope using identical illumination and magnification. Digital planimetry was performed using ImageJ with fixed threshold settings to segment the pale TTC-negative area, which was expressed as a percentage of the total slice area. The results were averaged for each slice, corrected for slice mass and aggregated to yield % infarct size for the total ventricular mass. Image analysis was performed by an investigator blinded to group allocation.

In other experiments, the perfusion of the hearts was stopped at the indicated stages (control perfusion, ischemia-only, and I/R), and the hearts were immediately immersed in 4% PFA in KH buffer containing 1% KCl to maintain diastolic arrest, fixed overnight at room temperature, and subsequently processed for immunohistochemistry.

### Immunohistochemistry staining, confocal imaging, and analysis

Hearts were carefully extracted and post-fixed by immersion in 4% PFA overnight at room temperature. Following post-fixation, hearts were transferred to a 30% sucrose solution in PBS for cryoprotection and stored at 4°C until they sank to the bottom of the container, typically 24–48 h later. The heart was then embedded in Optimal Cutting Temperature compound, frozen on dry ice, and stored at −80°C until sectioning. Coronal sections (30 μm thick) were obtained using a Tanner TN60 cryostat maintained at −20°C. Sections were serially collected and stored in PBS supplemented with 0.1% sodium azide.

For antibody staining, sections were first blocked and permeabilized for 1 h at room temperature in a solution containing 10% NGS and 0.05% Triton X-100 in PBS. Following this step, sections were incubated with anti-G3BP1 (1:1000, Proteintech, Cat. No. 13057-2-AP, Host: Rabbit), diluted in 5% NGS in PBS-T overnight at 4°C with gentle orbital shaking. After primary antibody incubation, sections were washed four times with PBS-T to remove unbound antibodies. Following the washes, sections were incubated with Alexa Fluor 546 anti-rabbit IgG (Thermo Fisher, A11071, 1:1000). Incubations with secondary antibodies were performed at room temperature with gentle orbital shaking for 1 h, protected from light. This was followed by staining with Hoechst 33342 (1:5000 dilution from a 10 mg/ml stock solution, Cat. No. H3570) for 15 min in the dark. Sections were then thoroughly washed in PBS-T and mounted on glass slides using Vibrance anti-fading medium (Vector Laboratories). Images were acquired using the 100×/1.45 NA oil-immersion objective of the Olympus FV3000 confocal microscope equipped with 405, 488, and 561 nm laser lines. Multiple non-overlapping FOVs were collected from different anatomical positions of the heart, and at least three sections per heart were collected, quantified, and data averaged.

### Immunoprecipitation of EGFP-G3BP1 and O-GlcNAc site discovery

Cells were transduced with either a control β-galactosidase–expressing adenovirus or an adenovirus encoding EGFP–G3BP1 (MOI 50) and harvested 24 h post-transduction. All samples were lysed in IP buffer (150 mM NaCl, 1% (v/v) NP-40, 0.5 mM EDTA, protease and phosphatase inhibitors, 1 mM freshly added PMSF, and 2 μM Thiamet-G in 10 mM Tris–HCl, pH 7.6), followed by clarification at 21 300 × ***g*** for 15 min at 4°C. Lysates were then pre-cleared by end-over-end rotation for 1 h with NAR to remove proteins binding non-specifically to the matrix prior to immunoprecipitation with GFP-Trap resin (alpaca nanobody conjugated to agarose resin; Cat. No. gta-10, Proteintech).

To assess non-specific binding, control IP reactions were set up using harvesting buffer alone incubated with GFP-Trap resin, as well as pooled lysate incubated with NAR. Five hundred micrograms of total protein were incubated with 25 μl of GFP-Trap agarose slurry (1:1 resin suspension; Cat. No. gta-10, Proteintech) overnight at 4°C with end-over-end rotation. During the IP procedure, 2% of the input, flow-through, and final wash fractions were collected in parallel to evaluate pulldown efficiency and specificity by western blot. Beads were then washed five times with 450 μl wash buffer (150 mM NaCl, 1% (v/v) NP-40, 0.5 mM EDTA in 10 mM Tris–HCl, pH 7.6).

Bound proteins were eluted by boiling the resin in 1× LDS sample buffer (Invitrogen) supplemented with 50 mM DTT, RIPA buffer, and protease/phosphatase inhibitors at 95°C for 10 min. The resin was pelleted by centrifugation at 1 500 × ***g*** for 3 min, and the supernatant was collected for SDS–PAGE. Elution fractions, together with the previously collected input, flow-through, and wash samples, were analyzed by western blot using an anti-G3BP1 antibody (Cat. No. 13057-2-AP, Proteintech). Following quality assessment, the eluate was resolved on a 4%–12% Bis–Tris gradient gel and stained with SimplyBlue SafeStain (Invitrogen). The band corresponding to EGFP–G3BP1 (molecular mass: 79.1 kDa) was excised and processed for O-GlcNAc site mapping by mass spectrometry.

The excised gel band corresponding to EGFP–G3BP1 was subjected to in-gel trypsin digestion. Peptides were extracted and desalted using C18 reverse-phase and then separated by nano-flow reversed-phase liquid chromatography and analyzed on an Orbitrap Fusion Lumos mass spectrometer (Thermo Scientific) operating in data-dependent acquisition mode. An HCD-triggered EThcD acquisition strategy was employed: high-resolution HCD MS2 scans were first acquired in the Orbitrap and screened for the presence of diagnostic oxonium ions characteristic of O-GlcNAc modifications (m/z 204.0867, 186.0761, 168.0655, 144.0652, 138.0545, and 126.0550). Upon detection, EThcD fragmentation was triggered to enable confident site localization [118–120]. Raw data were analyzed using Proteome Discoverer (Thermo Scientific) with Mascot (Matrix Science) as the search engine and searched against the UniProt human proteome database (G3BP1 in the EGFP–G3BP1 construct was of human origin). Variable modifications included O-GlcNAc (HexNAc, +203.079 Da) on serine and threonine, oxidation (M), deamidation (NQ), and phosphorylation (S/T). A maximum of one missed cleavage was allowed. Precursor and fragment mass tolerances were set at 5 ppm and 10 ppm, respectively. O-GlcNAc site assignments were manually validated in Scaffold, based on the presence of diagnostic oxonium ions (e.g. m/z 204.0867, 186.0761, and 168.0655) and supportive fragment ion patterns when available, to ensure high-confidence localization.

### Bioorthogonal labeling of O-GlcNAc-modified proteins and detection of G3BP1 in hiPSC-CMs

JHU001 hiPSCs were generated from peripheral blood mononuclear cells obtained from a healthy 55–59-year-old White/Caucasian female donor by Sendai virus-mediated reprogramming [121]. JHU001 hiPSCs were differentiated into cardiomyocytes and maintained as previously described [122]. Briefly, hiPSCs were differentiated into cardiomyocytes using small-molecule modulation of Wnt signaling and used for experiments between 25 and 35 days post-differentiation. For bioorthogonal detection of O-GlcNAc-modified proteins, hiPSC-CMs were plated in 100 mm dishes at 5 × 10^6^ cells per dish and transduced with an adenovirus encoding human UAP1-F383G or with β-galactosidase control adenovirus at an MOI of 50 for 96 h. UAP1 catalyzes the conversion of GlcNAc-1-phosphate and UTP to UDP-GlcNAc. Previous studies have shown that substitution of UAP1 F383 with smaller residues reduces steric constraints within the substrate pocket and accommodates GlcNAc analogs bearing larger, click-compatible chemical handles [123,124]. The use of 1HexGlcNAlk as an alkyne-containing sugar analog for metabolic labeling of O-GlcNAcylated proteins has been described previously [125].

Transduced cells were incubated with 1HexGlcNAlk (50 μM, 16 h), were washed in ice-cold PBS (3×), lysed in a RIPA buffer containing 150 mM NaCl, 1.0% IGEPAL CA-630, 0.5% sodium deoxycholate, 0.1% SDS, and 50 mM Tris–HCl, pH 8.0, supplemented with 1 mM PMSF, 2 μM Thiamet-G, protease inhibitors, and phosphatase inhibitors. Lysates were sonicated and protein concentrations quantified using the bicinchoninic acid (BCA). Equal protein amounts from each sample (750 μg) were subjected to click-biotinylation in the presence of 2 mM sodium ascorbate, 100 μM THPTA, 1 mM CuSO_4_·5H_2_O, and 40 μM Biotin Azide Plus (Cat. No. CCT-1488, Click Chemistry Tools), as previously described [126]. Following the click reaction, proteins were precipitated by methanol/chloroform extraction and resuspended in 0.05% SDS, 50 mM Tris–HCl, pH 8.0. Ten percent of this suspension was retained as the input fraction, and the remaining protein suspension was incubated with high-capacity streptavidin agarose resin (100 μl settled resin; Cat. No. 2035, Thermo Fisher) overnight at 4°C with end-over-end rotation. The resin was then pelleted by centrifugation to collect the biotinylated fraction, washed five times with IP wash buffer (1% NP-40, 150 mM NaCl, 50 mM HEPES, pH 7.9), and eluted in western blot sample buffer containing 1× LDS and 50 mM DTT. Input and streptavidin pulldown fractions were resolved by gel electrophoresis and analyzed by immunoblotting for G3BP1. Global metabolic labeling was assessed by streptavidin blotting of equal protein inputs.

### Western blot analysis

Cells were washed with ice-cold PBS (3×) and lysed in RIPA buffer (150 mM NaCl, 1.0% IGEPAL CA-630, 0.5% sodium deoxycholate, 0.1% SDS, 50 mM Tris, pH 8.0, Cat. No. R0278, Sigma) supplemented with 2 μM TMG, 1 mM PMSF, 1× protease and phosphatase inhibitors (cOmplete, mini, EDTA-free, and PhosSTOP, respectively, Millipore, Sigma), 50 mM DTT, and 1× LDS sample buffer (Thermo Fisher Scientific, NP0008). For the subsequent western blot analysis, each lane was loaded with 10 μg of protein and run on NuPAGE Bis-Tris 4% to 12% gradient gels (Thermo Fisher Scientific, Cat. No. WG1403). Following electrophoresis, proteins were transferred onto nitrocellulose membranes (Thermo Fisher Scientific, iBlot2 transfer stacks, Cat. No. IB23001). The membranes were stained using REVERT 700 Total Protein Stain (LI-COR, Cat. No. 926-11010) to visualize the total protein loading and blocked in 5% bovine serum albumin (BSA, Cat. No. 9998, Cell Signaling) in TBS for one hour at room temperature. Primary antibodies were diluted in 5% BSA and 0.1% Tween 20 in TBS, as indicated for each experiment, and incubated with membranes overnight at 4°C. Post-incubation, the membranes were thoroughly washed with TBS-T. Subsequently, IR dye-conjugated host-specific secondary antibodies were applied (dilution of 5000:1 in 5% BSA and 0.1% Tween20-TBS). The specific antibodies used in the present study are detailed in Supplementary Table S3. Detection and quantification of band intensities were accomplished using the Odyssey system and Image Studio Lite software (LI-COR). Normalization for protein loading was achieved by comparing the band intensities to the total protein loading.

### GFP-Trap pulldown and western blot analysis of G3BP1-associated O-GlcNAc signal

For G3BP1 O-GlcNAc immunoblotting experiments, cells were transduced with adenoviruses encoding β-galactosidase (β-gal) control, WT EGFP–G3BP1, or T268A EGFP–G3BP1, as indicated, and harvested after EGFP–G3BP1 expression was confirmed by fluorescence microscopy. Samples were processed using the same GFP-Trap pulldown workflow described above. For sodium arsenite stress experiments, β-gal-transduced control cells and cells expressing WT EGFP–G3BP1 were left untreated or treated with sodium arsenite (500 μM) for 30 or 60 min before lysis and pulldown, as indicated. Input, eluate, and flow-through fractions were resolved by SDS–PAGE and analyzed by western blotting. To assess G3BP1-associated O-GlcNAc signal, membranes containing the same samples were sequentially probed for O-GlcNAc and G3BP1 using reciprocal antibody combinations. In one approach, O-GlcNAc was detected using RL2, an in-house mouse anti-O-GlcNAc antibody (500:1), and G3BP1 was detected using a rabbit anti-G3BP1 antibody (Proteintech, 13057-2-AP; 2000:1). In the reciprocal approach, O-GlcNAc was detected using a rabbit anti-O-GlcNAc multiMAb (Cat. No. 82332; 500:1), and G3BP1 was detected using a mouse anti-G3BP1 antibody (Proteintech, 66486-1-Ig; 3000:1).

### Cell viability assays

To validate the protective effect of stress granules, four-chamber ibidi slides were utilized. After seeding, cells were allowed to adhere and proliferate in CGM for 24 h. At this point, the medium was replaced with fresh CGM to maintain nutrient levels and optimal conditions for cell growth. At 48 h post-passaging, two wells of the slide were treated with 500 μM sodium arsenite for 30 min to induce cellular stress, while the remaining two wells served as controls and were treated with CGM alone. Following this initial treatment, all medium was carefully removed. One well from the sodium arsenite-treated group and one well from the control group were exposed to 10 nM staurosporine for 1 h to induce apoptosis. The other two wells were treated with a 1:1000 dilution of dimethyl sulfoxide (DMSO) in CGM as a vehicle control. During the final 15 min of the incubation period, cells were counterstained with NucBlue to visualize nuclei. The staining allowed accurate identification of apoptotic and viable cells during imaging. Following the incubation and staining processes, cells were imaged to capture treatment-specific cellular responses.

Gene knockdown experiments were conducted using eight-chamber ibidi μ-slides to evaluate the functional impact of specific gene silencing on cell viability under stress conditions. To facilitate comparative analysis, the top row of the eight-chamber slide was designated as the NTC group, while the bottom row contained cells transfected with siRNAs targeting OGT or G3BP1/2. Once transfection was complete, the experimental procedures performed in the four-chamber ibidi slides, including sodium arsenite and staurosporine treatments, were replicated for the NTC and siRNA-transfected groups, allowing direct comparisons of treatment effects between groups.

Cellular imaging was performed using the Invitrogen™ EVOS™ XL Core Imaging System, equipped with 20× phase contrast and fluorescence microscopy capabilities. For each chamber, three distinct fields of view were selected to ensure representative sampling of both phase contrast and fluorescent micrographs. Images captured included a diversity of cell populations and morphological features to account for spatial heterogeneity. Fluorescent micrographs were utilized to identify nuclei stained with NucBlue, facilitating differentiation between apoptotic and viable cells. Image analysis was conducted using ImageJ software, which allowed precise quantification of condensed nuclei, a hallmark of apoptosis. The total number of apoptotic cells identified was divided by the total number of nuclei to calculate the proportion of viable cells following each treatment. This metric provided a quantitative measure of cell survival and enabled robust comparisons across experimental conditions.

### Statistical analysis

Data were handled and plotted using Microsoft Excel and GraphPad Prism. Values in the graphs are reported as means ± standard error. The numbers of biological replicates are shown inscribed in their respective bars or on the side of curves. Data were examined for normality of distribution using the Shapiro-Wilk test. To identify statistically significant differences between two groups, we performed unpaired Student’s t-test, unless otherwise stated in the figure legend. When comparing three or more groups, we used one-way or two-way ANOVA depending on the number of factors (e.g. for comparing the effect across 3 or more drugs, we used one-way, whereas for comparing groups resulting from a combination of drugs across several time points, we used two-way). To identify statistically significant differences, the data were assessed for normality of distribution. If the majority of groups within an experiment had normally distributed data, we used the Tukey *post-hoc* test. If the distribution of the data in the majority of groups was not normal, we used the Dunnett *post-hoc* test. In all cases, the α level for statistical significance was 0.05. *P* values less than 0.05, 0.01, 0.001, and 0.0001 are symbolized as *, **, ***, and **** respectively.

## Supplementary Material

Supplementary Figures S1-S10

Supplementary Table S1-S3

## Data Availability

All data supporting the conclusions of this study are included in the article and its Supplementary Information. Numerical source data underlying the reported quantitative analyses are available in a public data repository at 10.6084/m9.figshare.33652969. The mass spectrometry proteomics data have been deposited to the ProteomeXchange Consortium via the PRIDE partner repository with the dataset identifier PXD083651.

## Open Access

Open access for this article was enabled by the participation of Johns Hopkins University in an all-inclusive Read & Publish agreement with Portland Press and the Biochemical Society.

## Abbreviations

EGFP-G3BP1: N-terminal EGFP-tagged human G3BP1 (labeled GFP-G3BP1 in some figures for brevity)
FOV: field of view
HS: harvesting solution
hnRNPs: heterogeneous nuclear ribonucleoproteins
IDRs: intrinsically disordered regions
I/R: ischemia/reperfusion
MOI: multiplicity of infection
NRVMs: neonatal rat ventricular myocytes
NTC: non-targeting control
NTF2L: NTF2-like
NAR: neutral agarose resin
OGA: O-GlcNAcase
OGT: O-GlcNAc transferase
RGG: arginine-glycine-glycine
ROIs: Regions of interest
RRM: RNA-recognition motif
TTC: triphenyl-tetrazolium chloride
WT: wild-type
