# Supplementary material for: Loss of O-GlcNAc transferase activity impairs the dynamic formation of protective stress granules in ischemic cardiomyocytes
Source: Biochem J. 2026 Sep 16;483(10):1947–77. doi: 10.1042/BCJ20250300 (PMC13591266; doi:10.1042/BCJ20250300)
Supplement: Supplementary file 1 [file BCJ-2025-0300_supp.pdf]

Loss of O-GlcNAc Transferase activity impairs the dynamic formation of protective stress granules in ischemic cardiomyocytes

Hannah C. Swimm, Wenxi Zhang, Yashas Mallikarjun, Rohit Satish, Andres J. Medina, Sabrina Lo, Hisham Qureshi, Aidan J. Dunphy, Deepthi Ashok, Agnes Sidor, Olurotimi O. Mesubi, Brian O'Rourke, D. Brian Foster, Natasha E. Zachara & Kyriakos N. Papanicolaou

Supplemental material

Supplemental Figures 1-10

Supplemental Table 3

Suppl. Figure 1

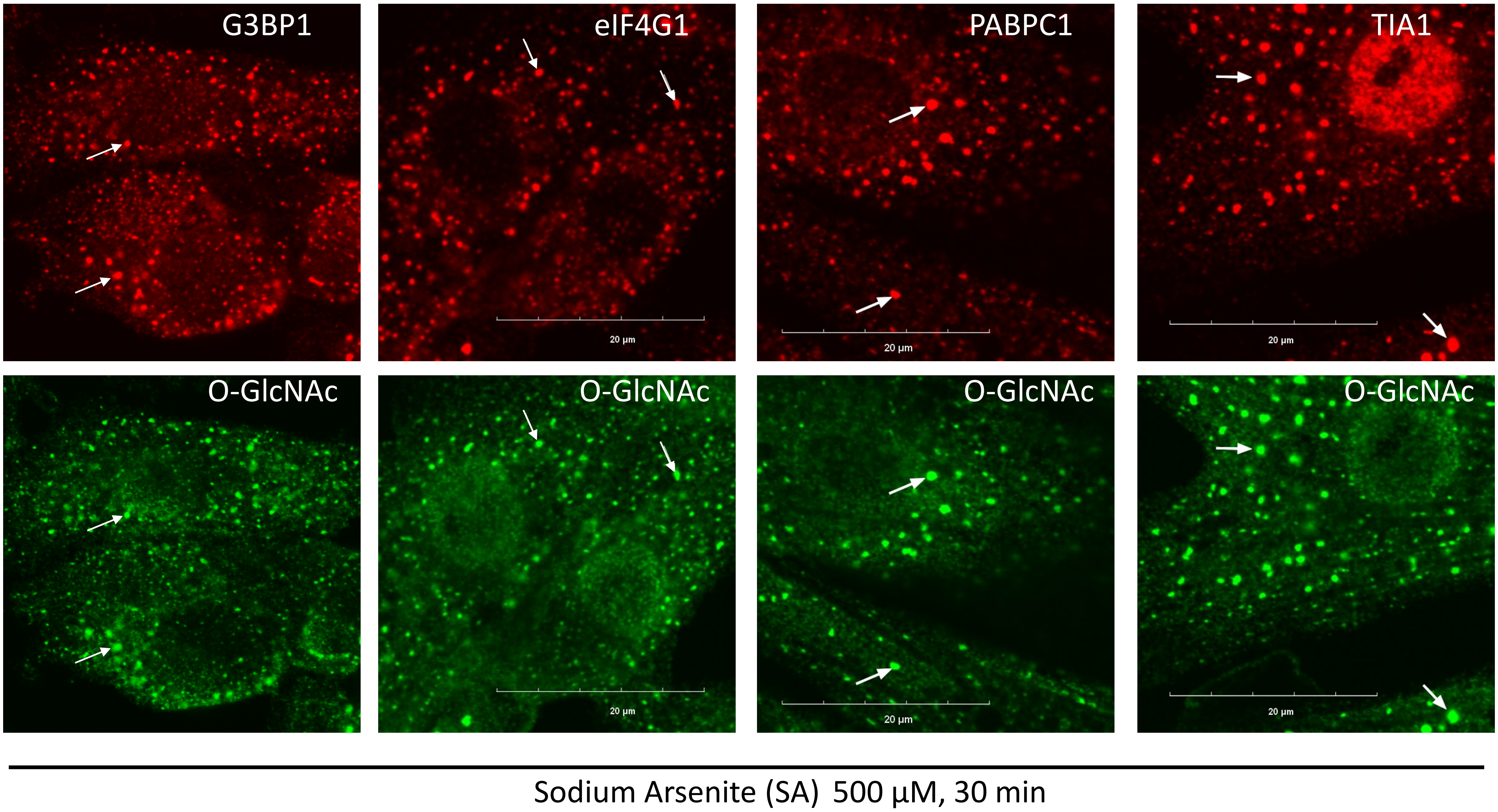

Supplemental Figure 1. Immunocytochemistry of stress granule markers and O-GlcNAcylation in cardiomyocytes. Primary neonatal ventricular myocytes (NRVMs) were seeded in ibidi chamber slides (2.5 × 10^5 cells/ chamber). To induce stress granule formation, cells were exposed to 500 µM and 30 min. later were fixed (4% pre-warmed paraformaldehyde in PBS), permeabilized and processed for dual immunocytochemistry for one of the following stress granule markers G3BP1, eIF4G1, PABPC1 and TIA1 (all raised in rabbit and used at a dilution 200:1) in combination with RL2 O-GlcNAc antibody (raised in mouse, used at a dilution 200:1). Secondary antibodies were anti-mouse Alexa Fluor 488 and anti-rabbit Alexa Fluor 546. Images were acquired on an Olympus FV3000RS confocal system using the 100× objective. Arrows indicate selected stress granules (~1 µm condensates in diameter) showing strong dual signals for all the stress granule marker and O-GlcNAc. Scale bar, 20 µm.

Suppl. Figure 2

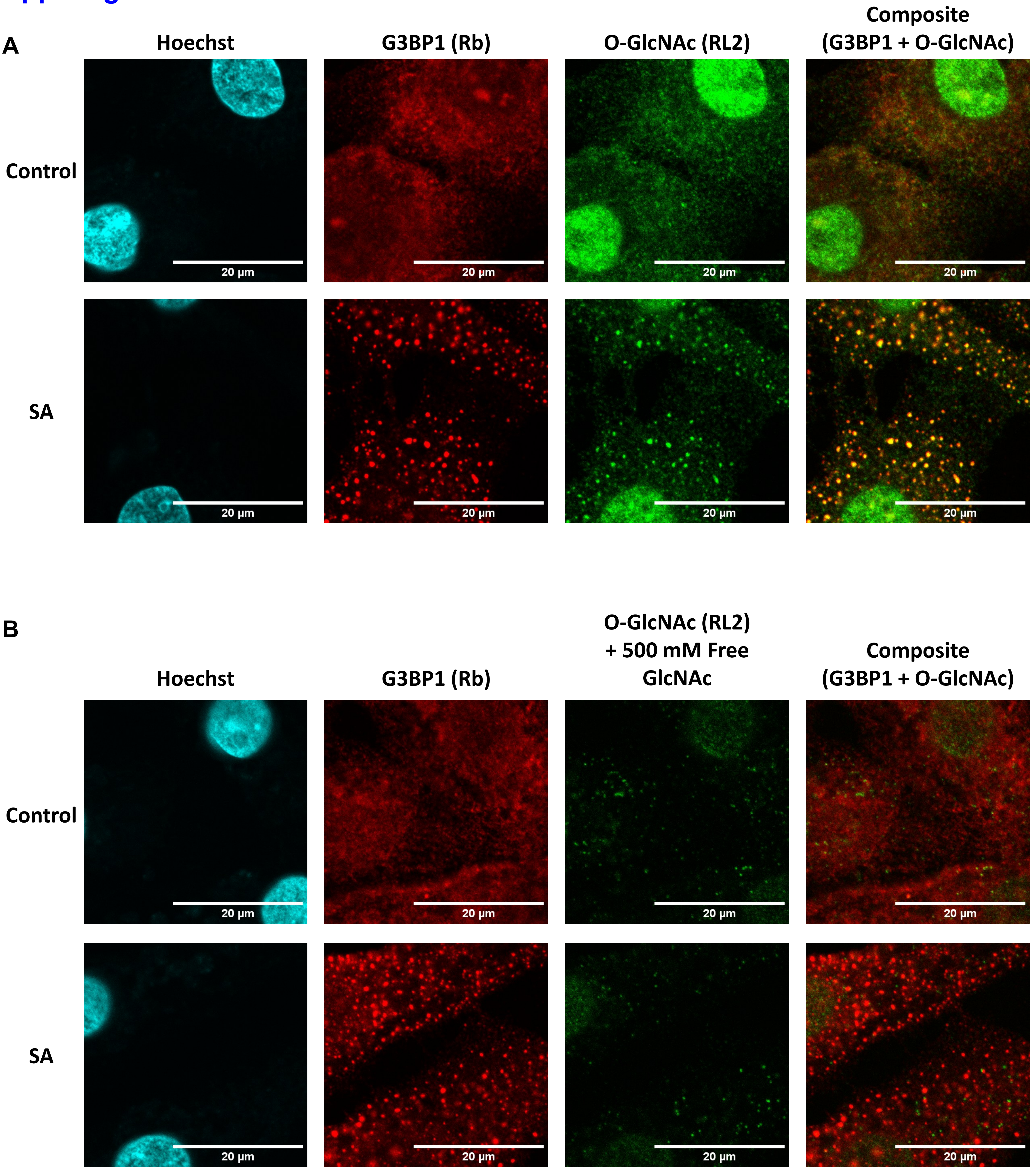

Suppl. Figure 2, continued

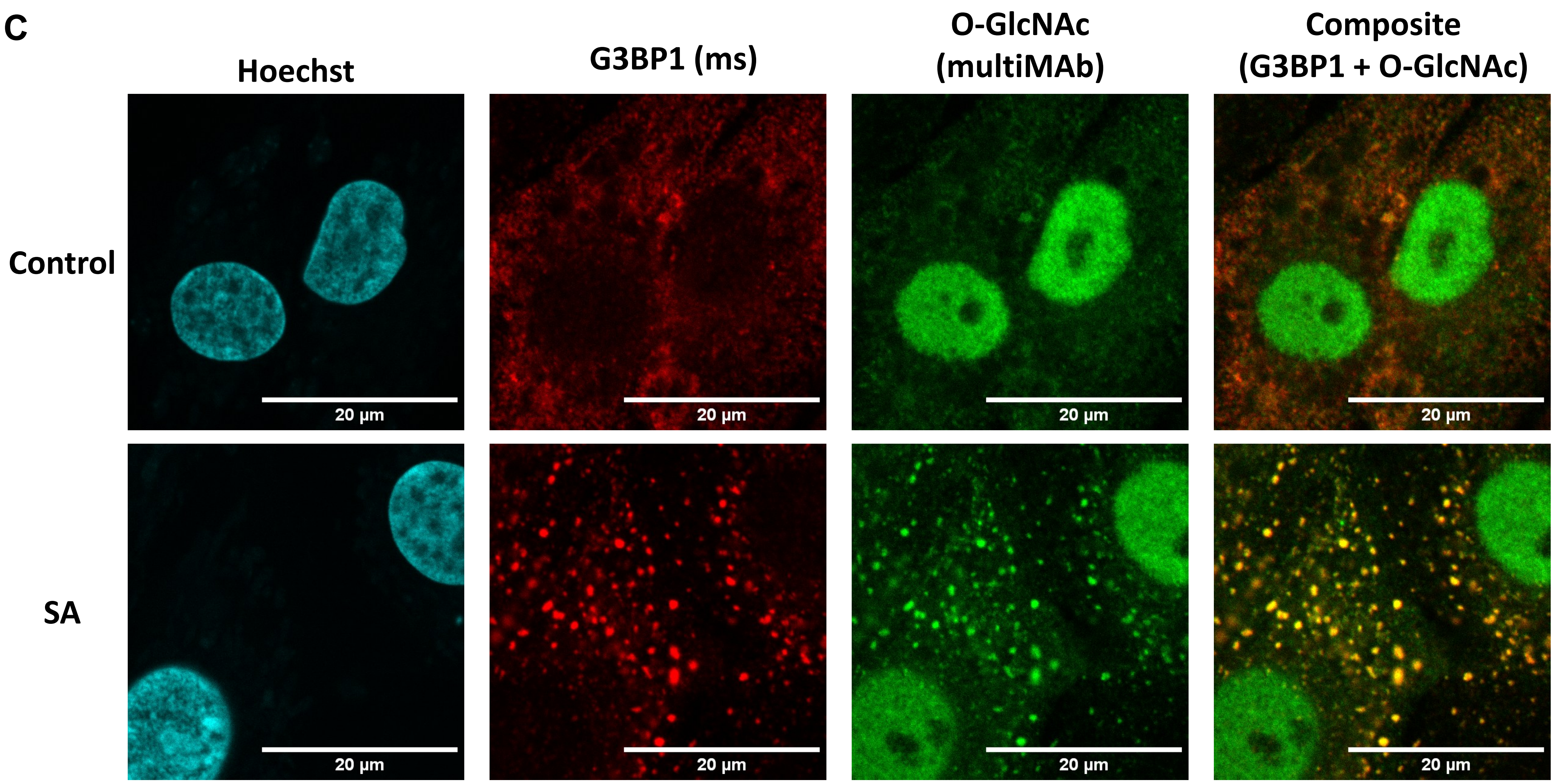

Supplemental Figure 2. Validation of O-GlcNAc immunoreactivity in stress granules.
(A) Representative immunofluorescence images of NRVMs stained with anti-G3BP1 (raised in rabbit; used at a dilution of 200:1; red) and anti-O-GlcNAc (RL2, raised in mouse; used at a dilution of 200:1; green) under basal conditions or following sodium arsenite treatment (SA; 500 µM, 30 min). Nuclei were counterstained with Hoechst (blue). Merged images demonstrate accumulation of O-GlcNAc immunoreactivity within G3BP1-positive SGs following SA treatment. **(B)** Representative immunofluorescence images of NRVMs stained as in (A), except that the RL2 antibody was preincubated with 500 mM free GlcNAc before application to cells. GlcNAc competition markedly reduced RL2 immunoreactivity while preserving G3BP1-positive SG formation following SA treatment. **(C)** Representative immunofluorescence images of NRVMs stained with anti-G3BP1 (raised in mouse; used at a dilution of 1000:1; red) and an independent anti-O-GlcNAc antibody (rabbit multiMAb; used at a dilution of 100:1; green) under basal conditions or following SA treatment. Similar accumulation of O-GlcNAc immunoreactivity within G3BP1-positive SGs was observed. Alexa Fluor 488- and Alexa Fluor 546-conjugated secondary antibodies were used for fluorescence detection. Images were acquired using a 100× objective. Scale bars, 20 µm.

Suppl. Figure 3

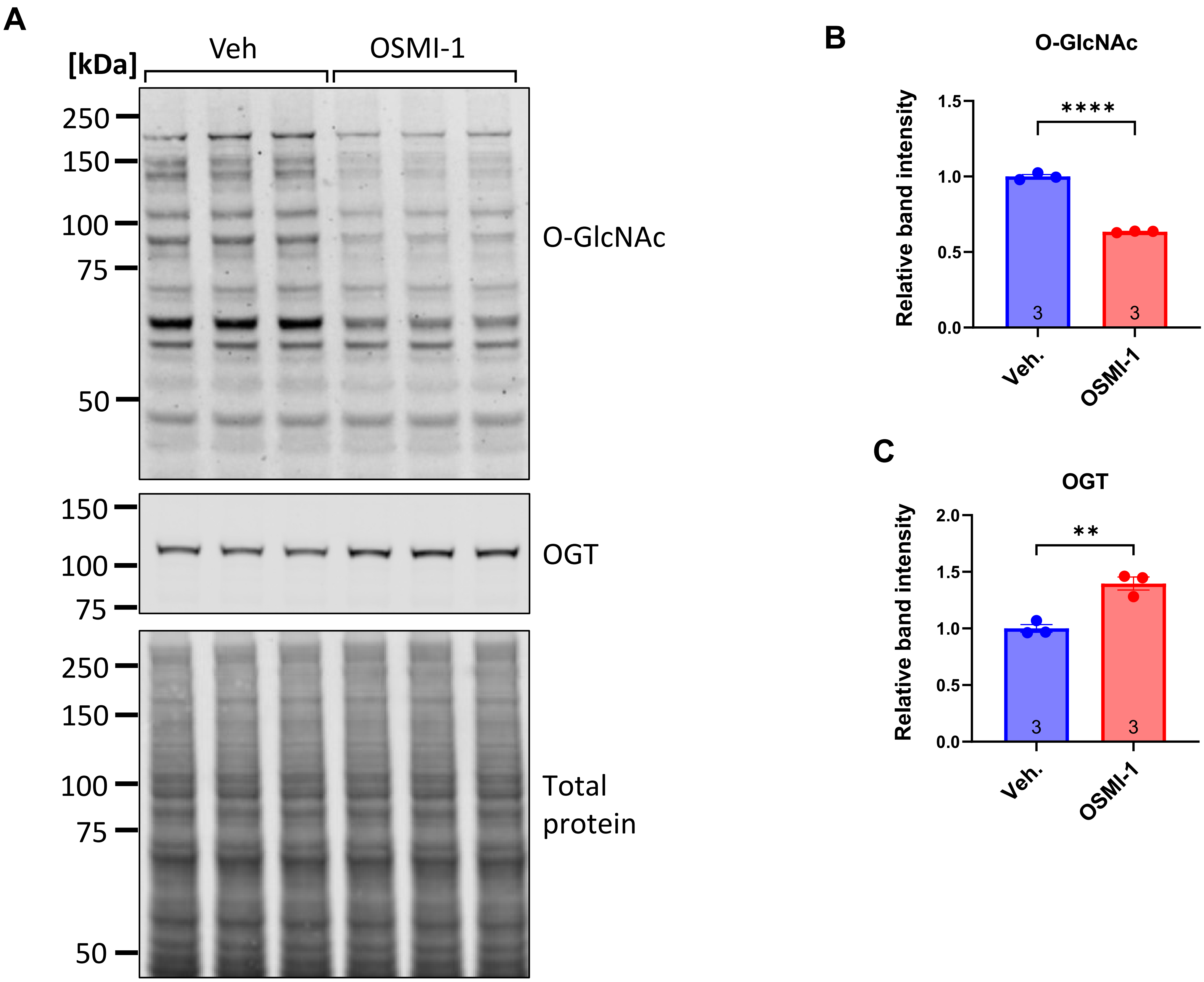

Supplemental Figure 3. Validation of pharmacologic OGT inhibition in H9c2 cells.
(A-C) Cells (1 × 10^6/well) were treated with Vehicle (0.1% v/v DMSO), or OSMI-1 (25 μM) for 6 h and were subsequently collected and proteins extracted for western blot for O-GlcNAcylation (antibody RL2, used at a dilution of 1000:1) and OGT (antibody AL24, used at a dilution of 4000:1). Total protein loading was assessed using Revert700 and used as normalizing loading control. Results of the densitometry for O-GlcNAc and OGT are shown in B and C respectively. Data are mean ± SEM from n = 3 biol. replicates/group. Comparisons were performed using an unpaired two-tailed t-test. ** $P < 0.01$, **** $P < 0.0001$

Suppl. Figure 4

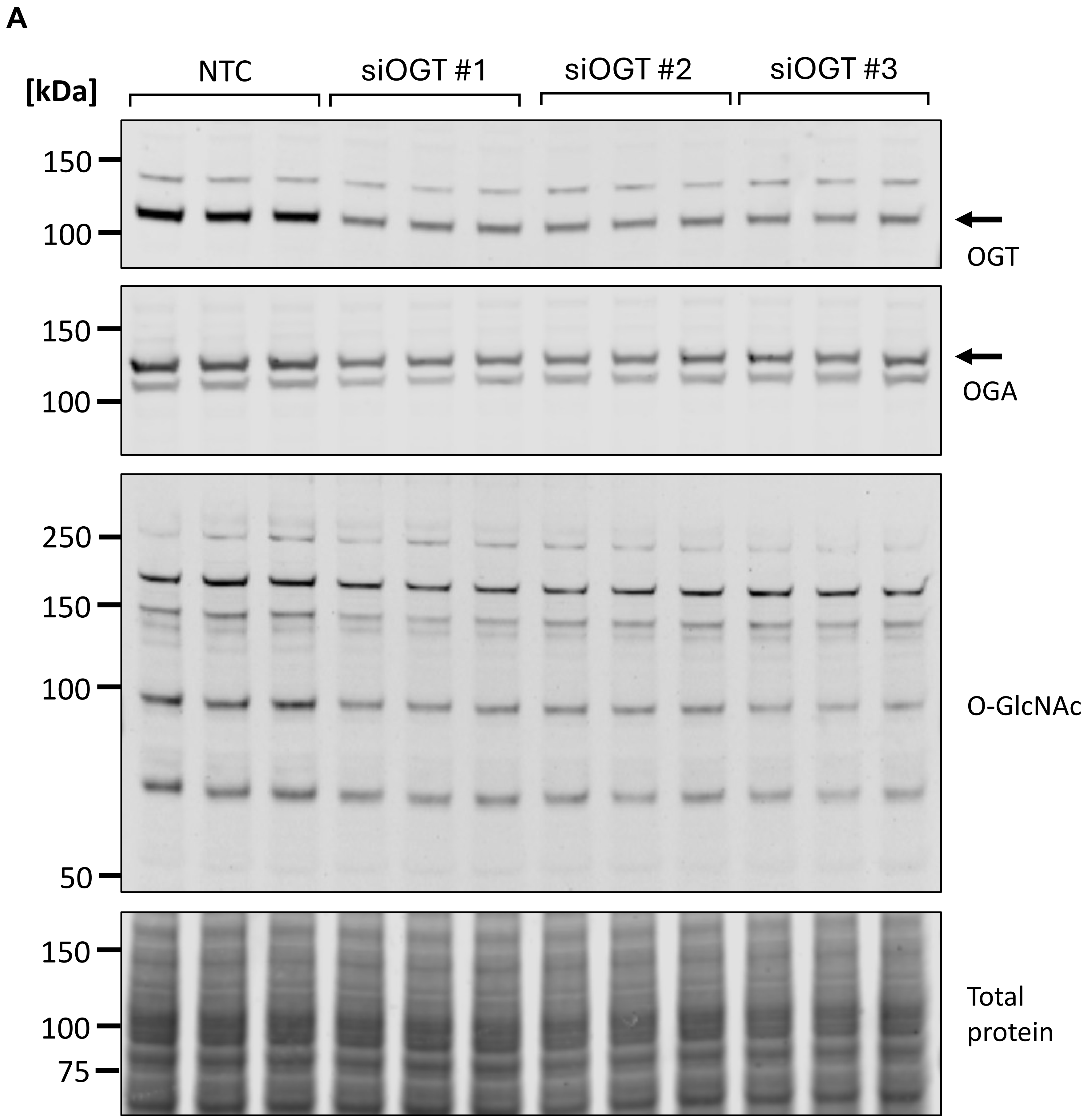

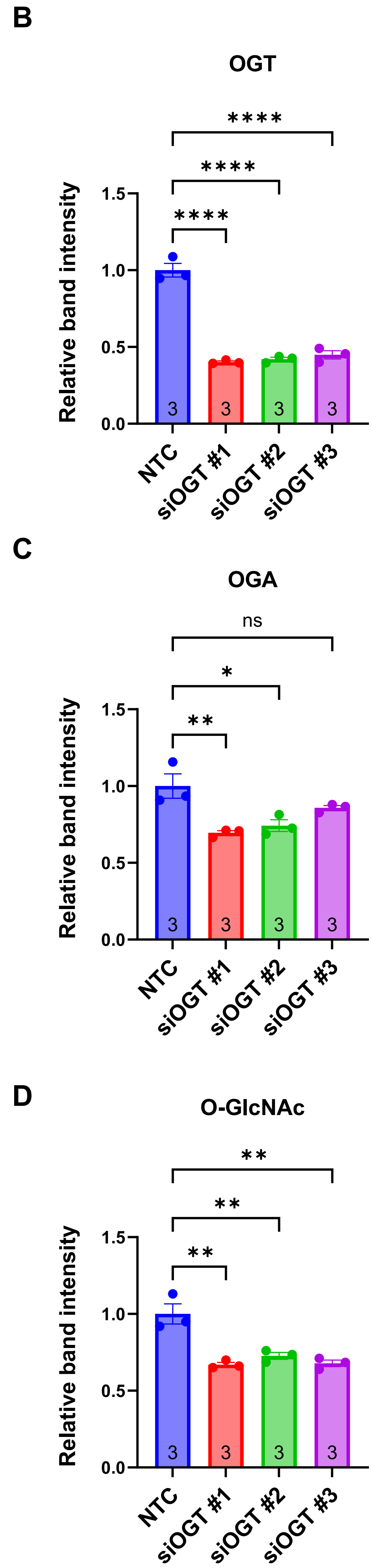

Supplemental Figure 4. Validation of OGT-targeting siRNAs in H9c2 cells.
(A) Representative immunoblots showing OGT, OGA, and global O-GlcNAc levels in cells transfected with a non-targeting control (NTC) or three independent OGT-targeting siRNAs (20 nM, 48 h). Total protein staining was used as a loading control. **(B-D)** Densitometric quantification of OGT (B), OGA (C), and global O-GlcNAc levels (D), normalized to total protein and expressed relative to the NTC condition. Data are presented as mean ± SEM from n = 3 independent biological replicates per group. OGT siRNA #1 was used for the OGT-KD condition shown in subsequent experiments. Statistical analysis was performed using one-way ANOVA followed by Tukey's multiple comparisons test. * $P < 0.05$, ** $P < 0.01$, **** $P < 0.0001$

Suppl. Figure 5

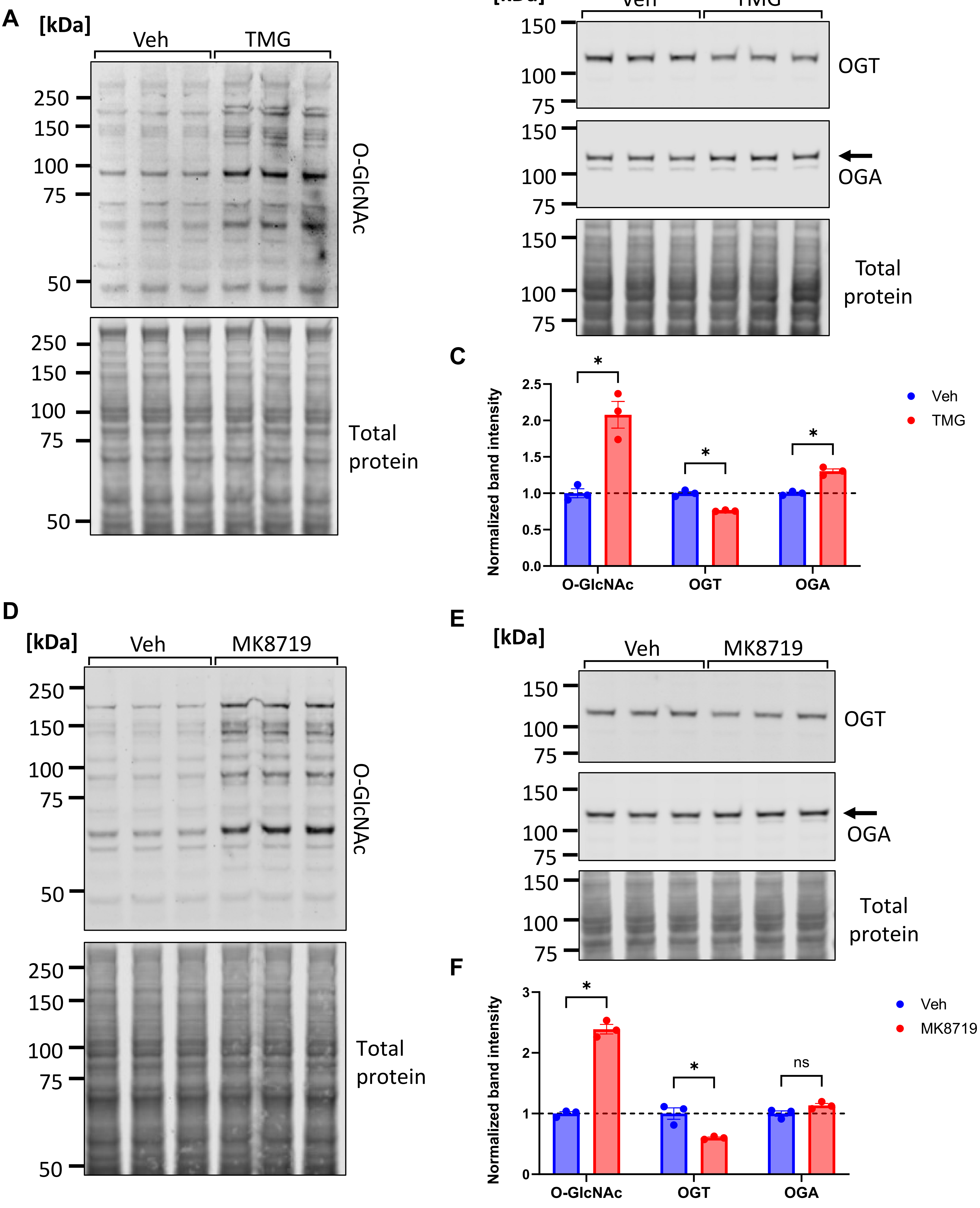

Suppl. Figure 5, continued

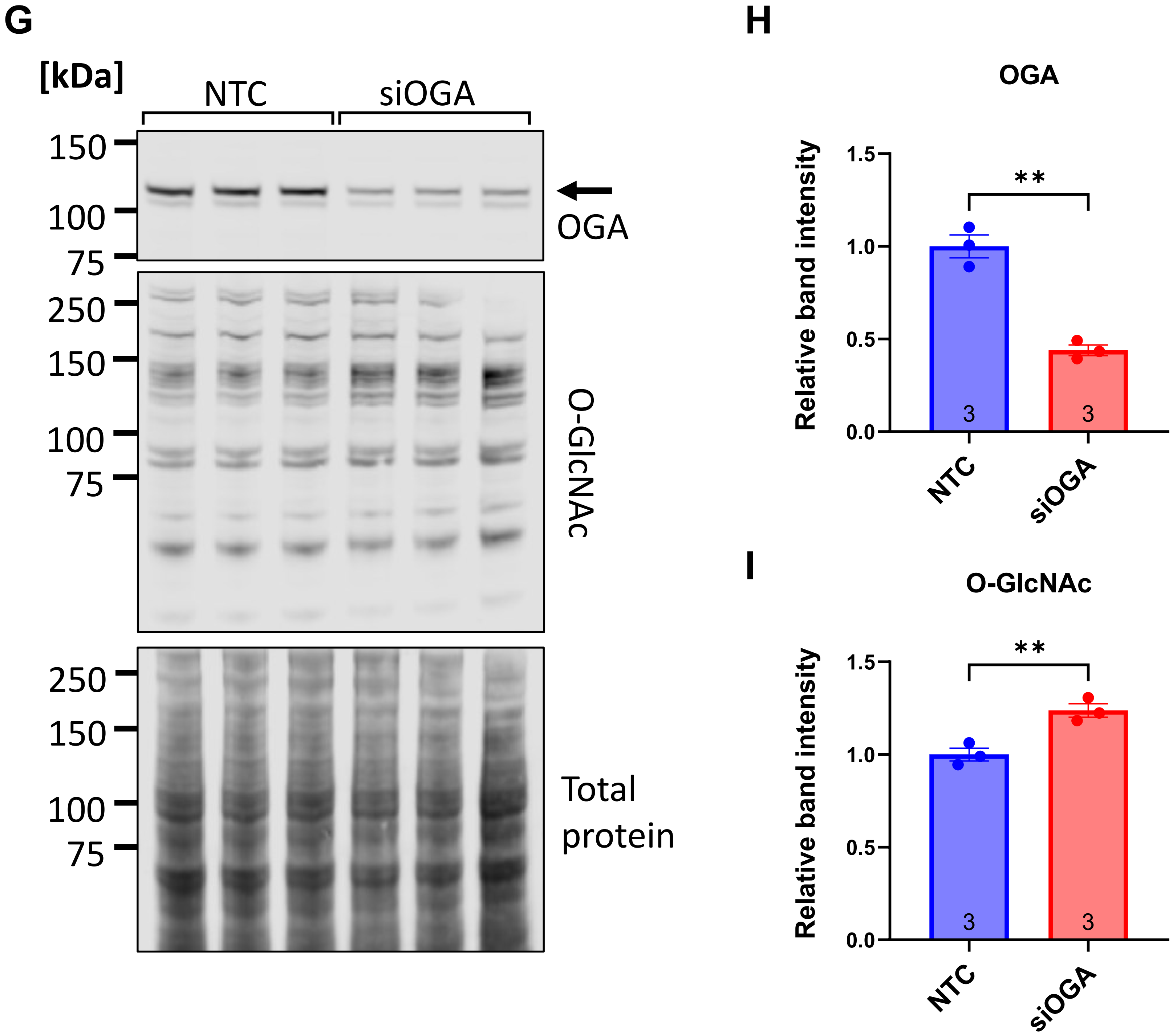

Supplemental Figure 5. Pharmacologic inhibition or genetic targeting of OGA increases cellular O-GlcNAcylation. **(A-C)** H9c2 cells were treated with vehicle (0.1% DMSO) or Thiamet-G (TMG; 25 µM, 6 h) and harvested for immunoblot analysis. Representative immunoblots showing global O-GlcNAc levels (A) and OGT and OGA abundance (B) are shown. Corresponding densitometric quantification of O-GlcNAc, OGT, and OGA levels is presented in (C). **(D-F)** H9c2 cells were treated with vehicle (0.1% DMSO) or MK8719 (25 µM, 6 h) and processed as above. Representative immunoblots showing global O-GlcNAc levels (D) and OGT and OGA abundance (E) are shown. Corresponding densitometric quantification of O-GlcNAc, OGT, and OGA levels is presented in (F). **(G-I)** H9c2 cells were transfected with a non-targeting control (NTC) or an OGA-targeting siRNA (20 nM, 48 h). Representative immunoblots showing OGA abundance and global O-GlcNAc levels are shown in (G), with corresponding densitometric quantification of OGA (H) and O-GlcNAc levels (I). Total protein staining was used as a loading control throughout. Data are presented as mean ± SEM from n = 3 independent biological replicates per group. Statistical comparisons for panels C and F were performed using unpaired two-tailed t-tests with Holm–Šídák correction for multiple comparisons. Statistical comparisons for panels H and I were performed using unpaired two-tailed t-tests. *P < 0.05, **P < 0.01, ***P < 0.001.

Suppl. Figure 6

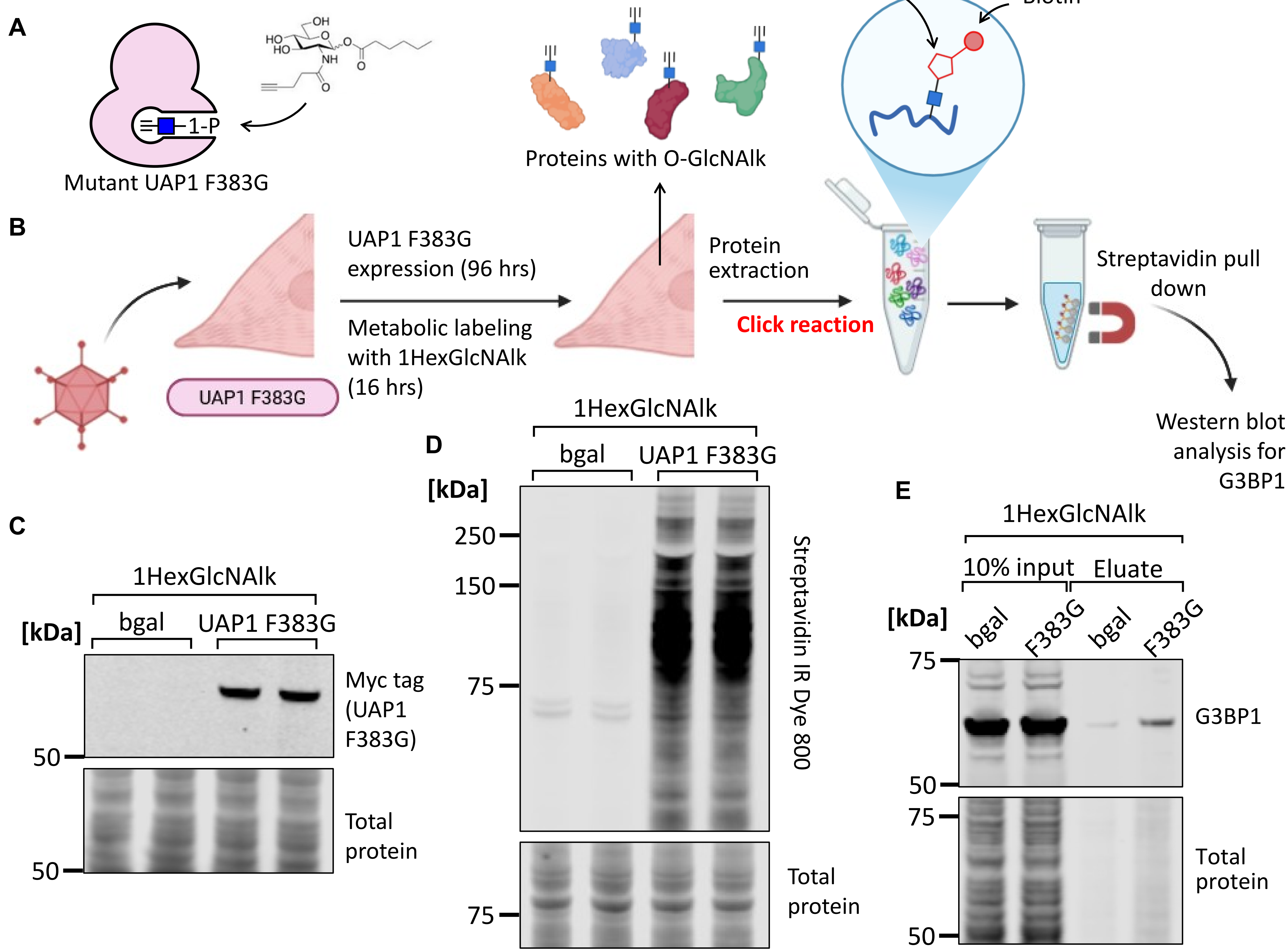

Supplemental Figure 6. Bioorthogonal metabolic labeling supports endogenous G3BP1 O-GlcNAcylation in hiPSC-derived cardiomyocytes.

(A) Schematic overview of the UAP1-F383G metabolic-labeling strategy. Substitution of UAP1 F383 with G, expands the substrate pocket and accommodates GlcNAc analogs bearing click-compatible chemical handles. The chemical structure of the alkyne-containing metabolic probe 1HexGlcNAlk is shown. **(B)** Experimental workflow for bioorthogonal detection of O-GlcNAc-modified proteins in hiPSC-derived cardiomyocytes. Cells were transduced with adenovirus encoding myc-tagged UAP1-F383G or β-galactosidase control and metabolically labeled with 1HexGlcNAlk. Following lysis, alkyne-labeled proteins were reacted with biotin-azide by click chemistry, forming a stable triazole linkage to a biotin-containing linker, shown schematically in the inset, and enriched by streptavidin pulldown prior to immunoblot analysis. **(C-D)** Validation of UAP1-F383G expression and global metabolic labeling. Immunoblotting for the myc tag confirmed expression of exogenous UAP1-F383G in UAP1-F383G-transduced cells but not in β-galactosidase control cells. Streptavidin blotting of equal protein inputs showed increased global click-biotinylated protein signal in UAP1-F383G-expressing cells. **(E)** Detection of endogenous G3BP1 in the streptavidin pulldown fraction following metabolic labeling and click-biotinylation. G3BP1 was detected in the streptavidin pulldown fraction from UAP1-F383G-expressing cells, supporting the presence of G3BP1 among metabolically labeled O-GlcNAc-modified proteins in hiPSC-derived cardiomyocytes.

Suppl. Figure 7

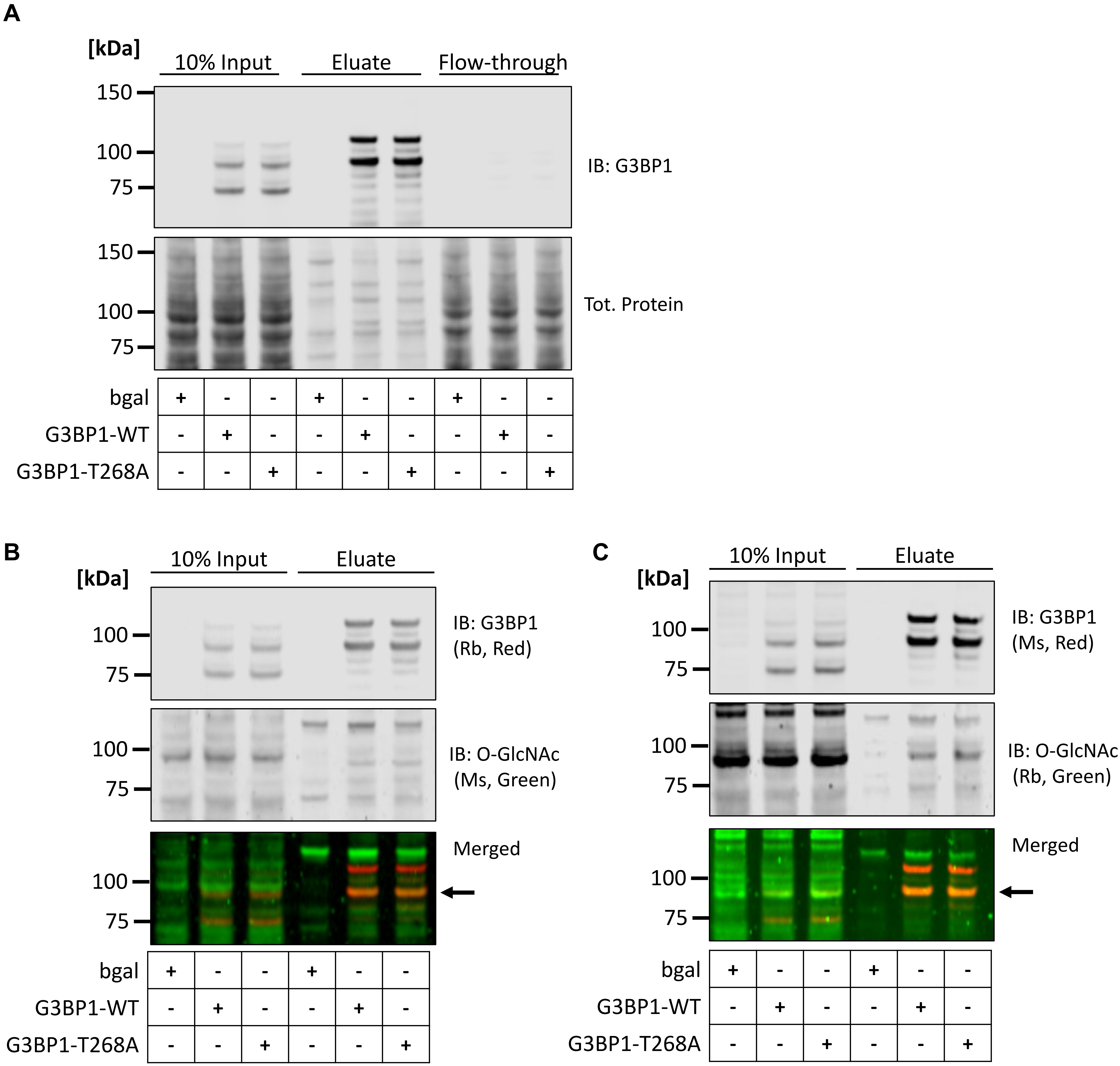

Suppl. Figure 7, continued

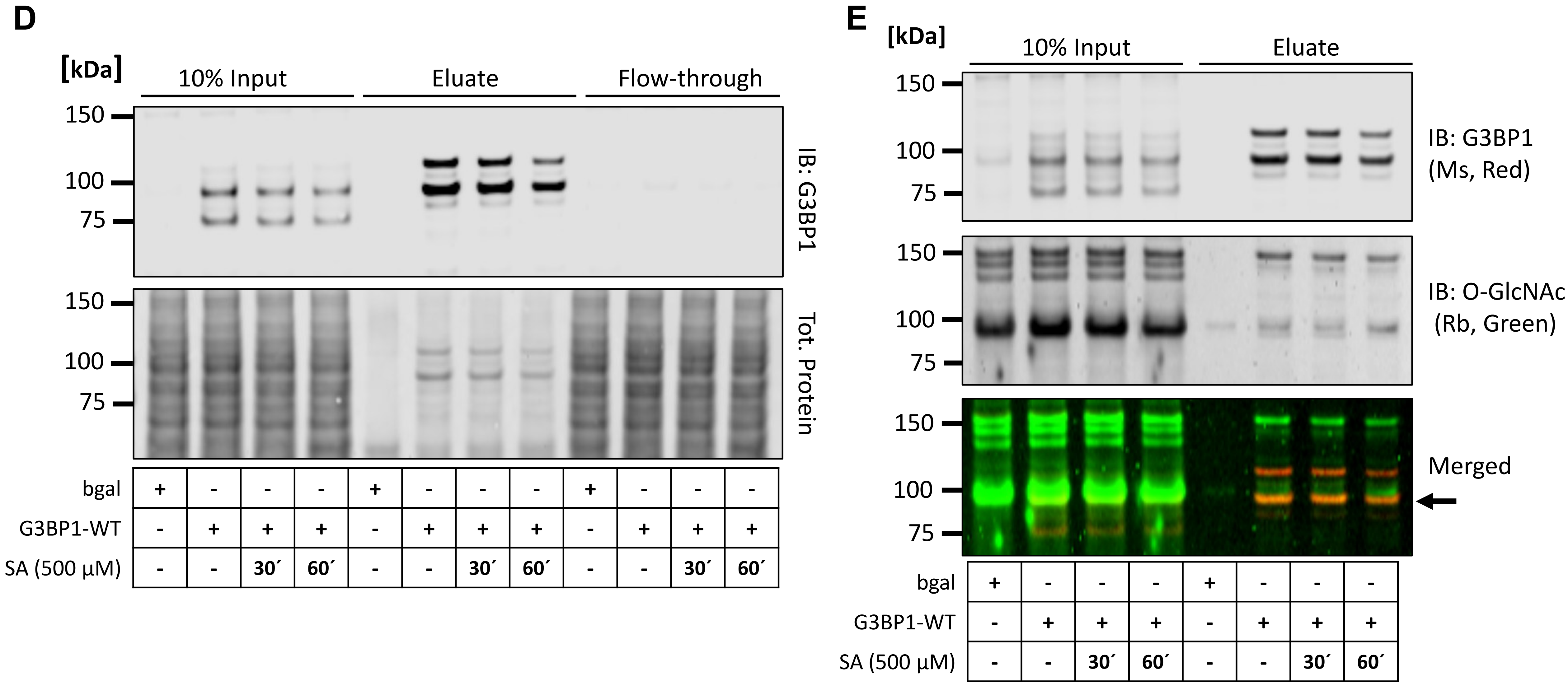

Supplementary Figure 7. Detection of G3BP1-associated O-GlcNAc immunoreactivity in WT, T268A, and sodium arsenite-treated samples. (A) Validation of G3BP1 enrichment by GFP-Trap pulldown. H9c2 cells were transduced with adenoviruses encoding β-galactosidase control (bgal) or the indicated WT and T268A G3BP1 constructs. Total protein lysates were subjected to GFP-trap affinity pulldown, and input, eluate, and flow-through fractions were analyzed by immunoblotting for G3BP1 using an antibody raised in rabbit (Proteintech, 13057-2-AP). WT and T268A G3BP1 were recovered in the eluate fractions, with minimal signal remaining in the flow-through. The bgal-transduced sample served as a negative control for enrichment. Total protein staining is shown below the G3BP1 immunoblot. **(B)** Detection of G3BP1-associated O-GlcNAc immunoreactivity using RL2. Input and eluate fractions from bgal, WT, and T268A samples were analyzed on the same membrane by sequential immunoblotting for O-GlcNAc using RL2, an antibody raised in mouse (green), and for G3BP1 using an antibody raised in rabbit (Proteintech, 13057-2-AP; red). O-GlcNAc immunoreactivity was detected in WT and T268A eluates, with little corresponding signal in the bgal control. The arrow in the merged blot indicates overlapping O-GlcNAc and G3BP1 signal at the enriched G3BP1 band. **(C)** Detection of G3BP1-associated O-GlcNAc immunoreactivity using a species-swapped antibody-detection strategy. The same samples were analyzed on the same membrane by sequential immunoblotting for O-GlcNAc using a rabbit anti-O-GlcNAc multiMAb (green) and for G3BP1 using an antibody raised in mouse (Proteintech, 66486-1-Ig; red). O-GlcNAc immunoreactivity was detected at the enriched G3BP1 band in WT and T268A eluates. Together, panels B and C provide support for O-GlcNAc reactivity at G3BP1 band and indicate that mutation of T268 does not abolish the total G3BP1-associated O-GlcNAc signal. **(D)** Validation of G3BP1 enrichment from untreated and sodium arsenite-treated cells. H9c2 cells expressing the WT G3BP1 construct were left untreated or treated with sodium arsenite for 30 or 60 min, as indicated, followed by GFP-trap affinity pulldown. Input, eluate, and flow-through fractions were analyzed by immunoblotting for G3BP1 using an antibody raised in rabbit (Proteintech, 13057-2-AP). Total protein staining is shown below the G3BP1 immunoblot. **(E)** Assessment of G3BP1-associated O-GlcNAc immunoreactivity under basal and stress conditions. Eluate fractions from untreated and sodium arsenite-treated cells were analyzed on the same membrane by sequential immunoblotting for O-GlcNAc using a rabbit anti-O-GlcNAc multiMAb (green) and for G3BP1 using an antibody raised in mouse (Proteintech, 66486-1-Ig; red). The arrow in the merged blot indicates overlapping O-GlcNAc and G3BP1 signal at the enriched G3BP1 band. Total immunoblot-detectable G3BP1-associated O-GlcNAc immunoreactivity did not show a stress-dependent increase under these conditions.

Suppl. Figure 8

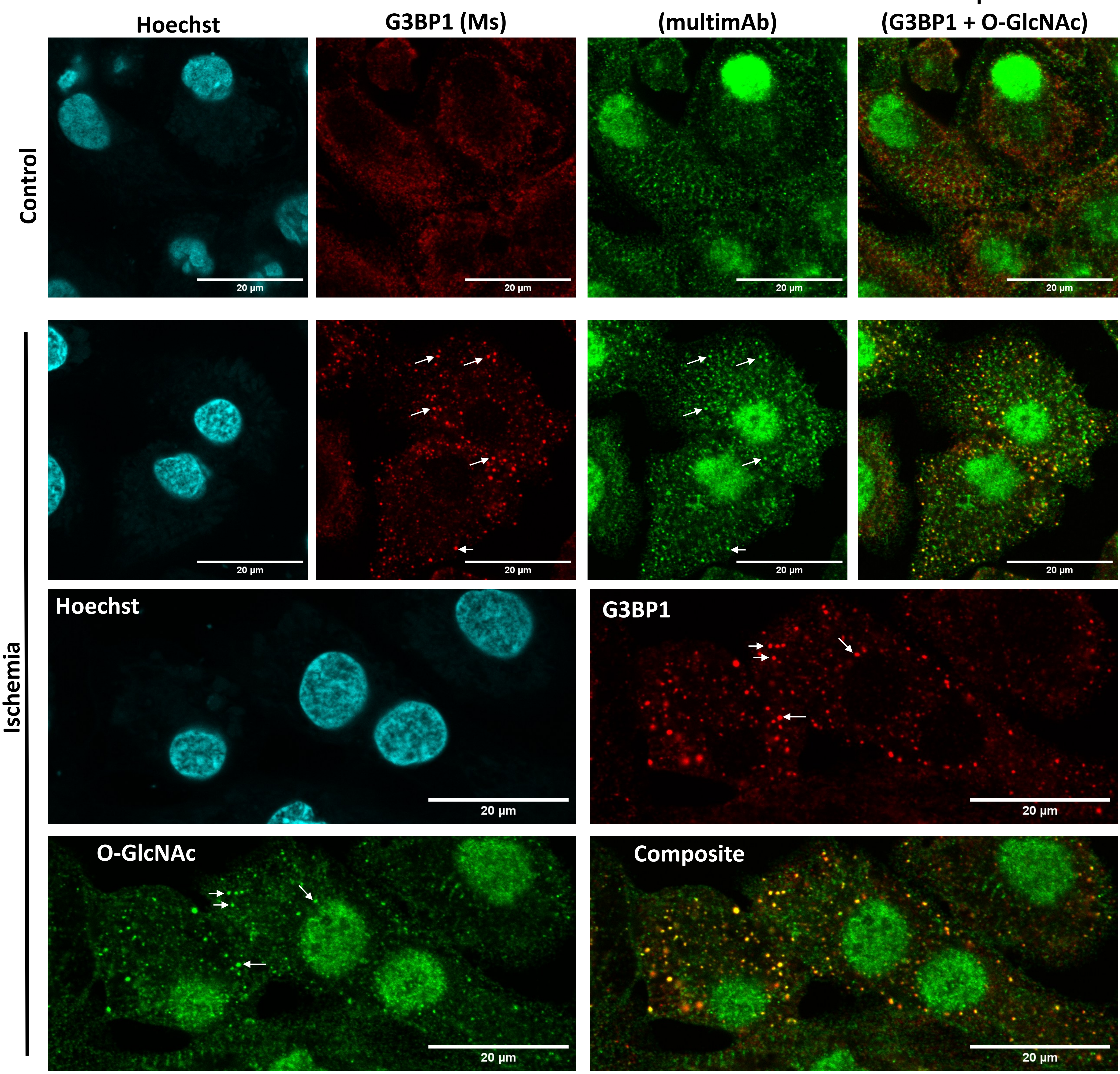

Supplemental Figure 8. O-GlcNAc immunoreactivity in ischemia-induced SGs using a second anti-O-GlcNAc antibody. Representative immunofluorescence images of NRVMs stained with anti-G3BP1 (raised in mouse; used at a dilution of 1000:1; red) and a second anti-O-GlcNAc antibody (rabbit multiMAb; used at a dilution of 100:1; green) under control conditions or following coverslip-induced ischemic stress. Ischemic stress was induced by placing a glass coverslip over the NRVM monolayer for 60 min, after which cells were fixed, stained, and imaged. Nuclei were counterstained with Hoechst (blue). Under ischemic conditions, G3BP1-positive SGs appear as spherical puncta that overlap with O-GlcNAc immunoreactivity, producing yellow puncta in the merged images. Arrows in the G3BP1 and O-GlcNAc channels indicate representative SGs with corresponding O-GlcNAc immunoreactivity. Scale bars, 20 µm.

Suppl. Figure 9

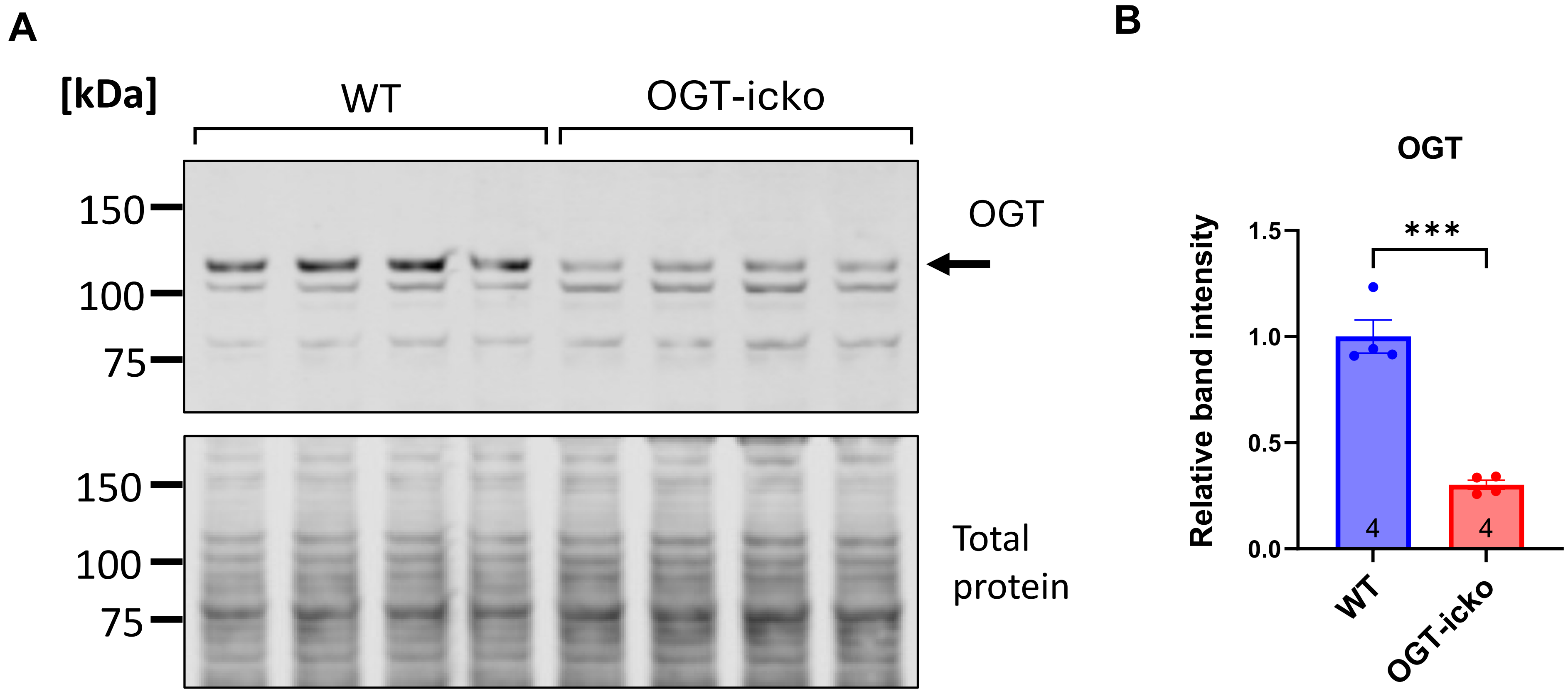

Supplemental Figure 9. Validation of OGT reduction in OGT-icko hearts.
(A) Representative immunoblot showing OGT abundance in whole-heart homogenates from wild-type (WT) and inducible cardiomyocyte-specific OGT knockout (OGT-icko) mice. OGT deletion was achieved using a tamoxifen-inducible Myh6-driven Cre recombinase system. Mice received four daily intraperitoneal injections of tamoxifen (20 mg/kg/day) and hearts were collected 10 days after the final injection. Total protein staining was used as a loading control. **(B)** Densitometric quantification of OGT abundance normalized to total protein and expressed relative to wild-type hearts. Data are presented as mean ± SEM from n = 4 WT or OGT-icko hearts. Statistical comparison was performed using an unpaired two-tailed t-test. ***P < 0.001

Suppl. Figure 10

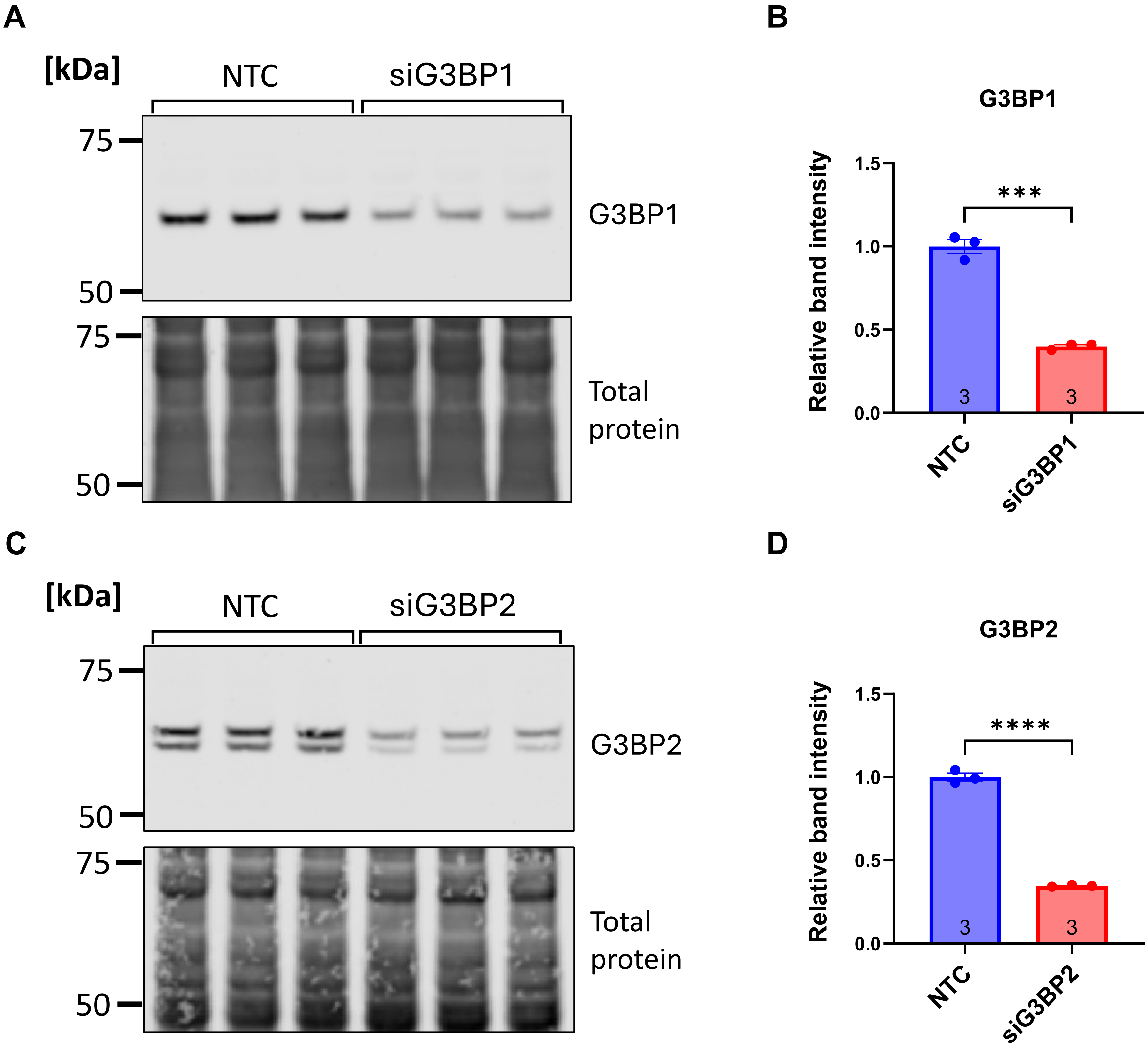

Supplemental Figure 10. Validation of G3BP1 and G3BP2 knockdown in H9c2 cells.
(A-B) Representative immunoblot showing G3BP1 abundance in cells transfected with a non-targeting control (NTC) or G3BP1-targeting siRNA (20 nM, 48 h). Total protein staining was used as a loading control. Corresponding densitometric quantification is shown in (B). **(C-D)** Representative immunoblot showing G3BP2 abundance in cells transfected with a non-targeting control (NTC) or G3BP2-targeting siRNA (20 nM, 48 h). Total protein staining was used as a loading control. Corresponding densitometric quantification is shown in (D). Data are presented as mean ± SEM from n = 3 independent biological replicates per group. Statistical comparisons were performed using unpaired two-tailed t-tests. ***$P < 0.001$, ****$P < 0.0001$.
