# Supplementary material for: Loss of O-GlcNAc transferase activity impairs the dynamic formation of protective stress granules in ischemic cardiomyocytes
Source: Biochem J. 2026 Sep 16;483(10):1947–77. doi: 10.1042/BCJ20250300 (PMC13591266; doi:10.1042/BCJ20250300)
Supplement: Supplementary file 2 [file BCJ-2025-0300_supp1.zip › BCJ-2025-0300-table3.pdf]

Supplemental Table 3. Reagent list

Antibodies		
**REAGENT**	**SOURCE**	**IDENTIFIER**
**Mouse monoclonal anti-O-GlcNAc**	Zachara Lab	RL2
**Rabbit anti-O-GlcNAc multiMAb**	Cell Signaling Technology	Cat# 82332<br>RRID: AB 2799991
**Rabbit monoclonal anti- MGEA5/OGA [EPR7154(B)]**	Abcam	Cat# ab124807,<br>RRID:AB_10971848
**Rabbit polyclonal anti- MGEA5/OGA**	Proteintech	Cat# 14711-1-AP,<br>RRID:AB_2143063
**Rabbit monoclonal anti- OGT (D1D8Q)**	Cell Signaling Technology	Cat# 24083,<br>RRID:AB_2716710
**Rabbit polyclonal anti- OGT**	Zachara Lab	AL24
**Rabbit polyclonal anti-G3BP1**	Proteintech	Cat# 13057-2-AP<br>RRID:AB_2232034
**Mouse monoclonal anti-G3BP1**	Proteintech	Cat# 66486-1-Ig<br>RRID:AB_2819031
**Rabbit monoclonal anti-eIF4G1**	Cell Signaling Technology	Cat# 2469<br>RRID:AB_2096028
**Rabbit polyclonal anti-PABPC1**	Proteintech	Cat# 10970-1-AP<br>RRID:AB_10596918
**Rabbit polyclonal anti-TIA1**	Proteintech	Cat# 12133-2-AP<br>RRID:AB_2201427
**Goat anti-Rabbit IgG Cross-Adsorbed Secondary Antibody, Alexa Fluor 546**	Thermo	Cat# A11071<br>RRID:AB_2534115
**Goat anti-Mouse IgG1 Cross-Adsorbed Secondary Antibody, Alexa Fluor 488**	Thermo	Cat# A21121<br>RRID:AB_2535764
**Goat anti-Rabbit IgG Cross-Adsorbed Secondary Antibody, Alexa Fluor 488**	Thermo	Cat# A11070<br>RRID:AB_2534114
**Goat anti-Mouse IgG Cross-Adsorbed Secondary Antibody, Alexa Fluor 546**	Thermo	Cat# A11030<br>RRID:AB_2737024
**IR Dye 800 Streptavidin**	LiCor	Cat# 926-32230 RRID not not listed
**Rabbit anti-myc tag antibody**	Cell Signaling Technology	Cat# 2272<br>RRID:AB_10692100

Animal strains		
**REAGENT**	**SOURCE**	**IDENTIFIER**
**OGT flox mice**<br>(B6.129-Ogttm1Gwh/J)	Jackson Laboratory	Stock No. 004860
**Myh6.MerCreMer transgenic mice**<br>(*A1cf*$^{Tg(Myh6\text{-}cre/Esr1^*)1Jmk}$/J)	Jackson Laboratory	Stock No. 005650
**Rosa26.tdTomato.Lox.Stop.Lox reporter mice**<br>B6.Cg-*Gt(ROSA)26Sor*$^{tm9(CAG\text{-}tdTomato)Hze}$/J or Ai9 (RCL-tdT)	Jackson Laboratory	Stock No. 007909

Chemicals and cell culture reagents		
**REAGENT**	**SOURCE**	**IDENTIFIER**
**OSMI-1**	Sigma-Aldrich	SML1621; CAS No.: 1681056-61-0
**Thiamet-G**	Tocris	Cat# 4390; CAS No.: 1009816-48-1
**$NaAsO_2$**	Sigma-Aldrich	S7400; CAS No.: 7784-46-5
**GlutaMAX supplement**	Thermo	Cat# 35050-061
**Fetal Bovine Serum (FBS)**	Thermo	Cat# A5670801
**Tamoxifen**	Sigma-Aldrich	T5648; CAS No.:10540-29-1
**Dimethyl sulfoxide**	Sigma-Aldrich	Cat# 472301; CAS No.: 67-68-5
**MK-8719**	MedChemExpress	Cat# HY-130121
**Paraformaldehyde**	Electron Microscopy Sciences	Cat# 15710
**Tritonx100**	Sigma-Aldrich	Cat# T9284
**Tween 20**	Promega	Cat# PRH5152
**Vibrance anti-fading medium**	Vector Laboratories	Cat# H-1700
**Penicillin Streptomycin**	Thermo	Cat# 15140-122
**HEPES**	Thermo	Cat# 15630-080
**Non Essential Amino Acids (NEAA)**	Thermo	Cat# 11140-050
**Fibronectin from bovine plasma**	Sigma Aldrich	Cat# F4759
**DMEM**	Corning	Cat# 10-013-CV
**Lipofectamine RNAiMax**	Thermo	Cat# 13778075
**Insulin Transferrin Selenium (ITS)**	Gibco	Cat# 51500056
**Bovine Serum Albumin (BSA)**	Cell Signaling Technology	Cat# 9998
**Normal Goat Serum (NGS)**	Cell Signaling Technology	Cat# 5425
**Optimal Cutting Temperature (OCT) compound**	Sakura	Cat# 4583
**RIPA buffer**	Sigma-Aldrich	Cat# R0278
**Phenylmethanesulfonyl fluoride**	Sigma-Aldrich	Cat# P7626
**cOmplete, Mini, EDTA-free Protease Inhibitor Cocktail**	Sigma-Aldrich	Cat# 11836170001
**PhosSTOP**	Sigma-Aldrich	Cat # 4906845001
**Dithiothreitol (DTT)**	Sigma-Aldrich	Cat# D9163-5G
**LDS sample buffer**	Thermo	Cat# NP0008
**Staurosporine**	Sigma Aldrich	Cat# S5921
**OPTI-MEM**	Thermo	Cat# 31985
**NucBlue Live Cell Stain ReadyProbe**	Thermo	Cat# R37605
**Hoechst 33342**	Thermo	Cat# H3570
**Free GlcNAc**	Sigma	Cat# A4106
**1HexGlcNAlk**	Tocris	Cat# 7751
**Pierce High Capacity Streptavidin Agarose**	Thermo	Cat# 20359
**Biotin Azide Plus**	Vector Laboratories	Cat# CCT-1488
**CuSO4**	Sigma	Cat# 469130
**THPTA**	Vector Laboratories	Cat# CCT-1010

Experimental models: Organisms/strains		
**REAGENT**	**SOURCE**	**IDENTIFIER**
**Timed pregnant female rats, outbred (for NRVM isolation)**	Envigo	Hsd:Sprague Dawley Rat
**H9c2 cells**	ATCC	Cat# CRL-1446
**hiPSC-CMs**	Chua CJ, DiSilvestre D, et al., 2025;88:103819	JHU001

Oligonucleotides		
**REAGENT**	**SOURCE**	**IDENTIFIER**
**Non targeting control (NTC)**	IDT DNA	51-01-14-04
**ds RNA sequence targeting rat OGT (rn.Ri.Ogt.13.2, referred in the text as siOGT#1)**<br>Top: AAA GCA GUC AUC GAU UUU AAA UCT A<br>Bottom: UAG AUU UAA AAU CGA UGA CUG CUU UUU	IDT DNA	Transcript:<br>NM_017107
**ds RNA sequence targeting rat OGT (rn.Ri.Ogt.131, referred in the text as siOGT#2)**<br>Top: CUU GAA UUC UUG AUG GUA AAA AAC A<br>Bottom: UGU UUU UUA CCA UCA AGA AUU CAA GCU	IDT DNA	Transcript:<br>NM_017107
**ds RNA sequence targeting rat OGT (rn.Ri.Ogt.13.3, referred in the text as siOGT#3)**<br>Top: GCC AAU CAC UUC AUU GAU CUU UCT C<br>Bottom: GAG AAA GAU CAA UGA AGU GAU UGG CUU	IDT DNA	Transcript:<br>NM_017107
**ds RNA sequence targeting rat OGA (rn.Ri.MGEA5.13.1)**<br>Top: CAA CUU CCC UUC UCU GAU AAA AAG<br>Bottom: CAU UUU UAU CAG AGA AGG GAA GUU GGC	IDT DNA	Transcript:<br>NM_131904<br>Targets exon 15
**ds RNA sequence targeting rat G3BP1 (rn.Ri.G3BP1.1.13.1)**<br>Top: GAC AUG UGA UUA CCA AAC AAU UCA<br>Bottom: UAG AAU UGU UUG GUA AUC ACA UGU CAA	IDT DNA	Transcript:<br>NM_133565<br>Targets exon 12
**ds RNA sequence targeting rat G3BP2 (rn.Ri.G3BP2.13.1)**<br>Top: CAG AGA AUC UUA AUU GCA AAA CCA<br>Bottom: UUG GUU UUG CAA UUA AGA UUC UCU GAA	IDT DNA	Transcript:<br>NM_001013989

Software and algorithms		
**REAGENT**	**SOURCE**	**IDENTIFIER**
**Fiji/ImageJ**	Schneider et al., 2012	https://imagej.nih.gov/ij/
**ImageStudio Lite**	LiCor	https://www.licor.com/bio/image-studio-lite/
**Graphpad Prism**	GraphPad Software	www.graphpad.com
**CellProfiler**	Stirling et al., 2021	https://cellprofiler.org/

Cell Culture Plates		
**REAGENT**	**SOURCE**	**IDENTIFIER**
**µ-Slide 4 well ibiTreat**	Ibidi	Cat# 80426
**µ-Slide 8 well ibiTreat**	Ibidi	Cat# 80806
**Glass bottom cell culture dish**	Nest	Cat# 801001
